# Implementation of SARS-CoV-2 genomic surveillance during the COVID-19 pandemic through an academic–public health collaboration in southeast Michigan

**DOI:** 10.1038/s41598-026-39974-7

**Published:** 2026-02-24

**Authors:** Rola Raychouni, Xiangmin Zhang, Samantha J. Bauer, Benjamin Wasinski, Katherine Gurdziel, Nivisa Vakeesan, Paige Stanton, Anthony T. Lagina, Michael Mossing, Geehan Suleyman, Jagjeet Kaur, Maryssa Trupiano, Phillip Levy, Paul E. Kilgore, Marcus Zervos, Steven Korzeniewski, Wanqing Liu

**Affiliations:** 1 Department of Pharmaceutical Sciences, Eugene Applebaum College of Pharmacy and Health Sciences, Wayne State University, Detroit, MI 48201 USA; 2 Department of Family Medicine and Public Health Sciences, Wayne State University, Detroit, MI 48201 USA; 3 Department of Emergency Medicine, Wayne State University, Detroit, MI 48201 USA; 4 Genome Sciences Core, Wayne State University, Detroit, MI 48202 USA; 5 Department of Pharmacology, School of Medicine, Wayne State University, Detroit, MI 48201 USA; 6 College of Health Sciences, Purdue University, West Lafayette, IN 47907 USA; 7 Department of Biochemistry, Microbiology, and Immunology, Wayne State University, Detroit, MI 48201 USA; 8 Department of Medicine, Division of Infectious Diseases, Henry Ford Health, Detroit, MI 48202 USA; 9 Department of Pharmacy Practice, Eugene Applebaum College of Pharmacy and Health Sciences, Wayne State University, Detroit, MI 48201 USA; 10 Department of Internal Medicine, Wayne State University, Detroit, MI 48201 USA; 11 Integrative Bioscience Center, 6135 Woodward Ave, Detroit, MI 48202 USA

**Keywords:** SARS-CoV-2, COVID-19, Genomic surveillance, Sequencing, Epidemiology, Computational biology and bioinformatics, Diseases, Genetics, Microbiology

## Abstract

Regional genomic surveillance is essential for tracking viral evolution and informing targeted public health responses. During the COVID-19 pandemic, we established a collaborative genomic surveillance pipeline for SARS-CoV-2 in Southeast Michigan to support national surveillance efforts and guide local pandemic response strategies. This work aims to present the methods and resources we have achieved during this effort and demonstrates the feasibility of such a collaboration. A partnership between Wayne State University (WSU), the Detroit Health Department (DHD), Henry Ford Health (HFH), the Wayne Health Mobile Unit (WHMU), and the Michigan Department of Health and Human Services (MDHHS) was established to collect, sequence, and analyze SARS-CoV-2 samples. Samples underwent automated nucleic acid extraction, RT-qPCR testing, and whole genomic sequencing at WSU’s integrative biosciences center (IBio). We analyzed consensus genome sequences using high-performance computing infrastructure for lineage assignment and variant identification. Between January 2022 and July 2024, we collected and archived 7508 samples, with 6235 (83.0%) successfully sequenced. A sub-analysis of 4637 HFH samples explored geographic distributions across 295 Michigan ZIP codes. Compared to the overall proportion of deaths among all people with SARS-CoV-2 positive tests in the sample (3.6% [95% CI (3.1, 4.2)]), the case-fatality rate was significantly increased with the 19 A + B [7.69%, 95% CI (5.03, 11.58)] and 20A (European 2 lineage: EU2) [9.65%, 95% CI (7.72, 11.99)] variants. The frequency distributions of variants showed a strong correlation (*r* = 0.98) with Michigan’s statewide data reported in GISAID. Omicron was the most prevalent variant detected (64% of cases). Our program demonstrated capacity for academic-public health partnerships to detect SARS-CoV-2 variant circulation in Southeast Michigan. This framework provides a replicable model for future pathogen surveillance programs to build on in response to infectious disease outbreaks.

**Supplementary Information:**

The online version contains supplementary material available at 10.1038/s41598-026-39974-7.

## Introduction

While the global COVID-19 pandemic necessitated overarching international guidance for pandemic management, regional investigation of viral spatiotemporal distribution and response efficacy in Southeast Michigan remained essential. During the first wave of the SARS-CoV-2 pandemic in the United States, the city of Detroit, Michigan, emerged as an early hot spot for related mortality^1^. By the 50th day of the virus reported in Michigan, more than one thousand COVID-19 related deaths were recorded amongst Detroiters; a per capita mortality rate [149 per 100,000 residents] that briefly eclipsed New York City as the global epicenter. Analyzing local pandemic patterns can reveal circulating lineages, assess transmission frequency, inform mitigation strategies, and develop precision responses tailored to this specific population. The significant impact of the pandemic highlighted the importance of building capacity to improve monitoring, preparedness, and response to outbreaks tailored to regional needs.

International experience has demonstrated that sampling, biobanking, and sequencing are essential for tracing outbreak origins, identifying vulnerable populations, and optimizing resource allocation^2^. Global and national driven consortiums managed the pandemic by developing centralized coordination through collaborative teams, setting benchmarks for sampling and turnaround times, linking metadata in consistent formats, rapid sharing of results, and sustaining funding^3–5^. In the United States, partnerships between academia, local public health agencies and health systems emerged to fill the void by working in collaboration with the U.S. Centers for Disease Control and Prevention (CDC)^6^. Consequently, lacking infrastructure for robust pathogen surveillance necessitated establishing a genomic surveillance program in the Detroit area.

Genomic surveillance is a highly effective approach for monitoring and analyzing genomic sequences to track variant spread and monitor SARS-CoV-2 genomic changes^7^. Viral genomic sequencing enabled virus identification and investigation of outbreak dynamics, while also informing the design of diagnostic assays, drugs, and vaccines. Through viral genomic sequencing as its foundation, genomic surveillance guided public health strategies, improved preparedness for future outbreaks, and contributed to countermeasure development as COVID-19 transitions to endemicity. This continuous monitoring facilitated epidemiological investigation and evaluation of control measure efficacy. Genomic surveillance achieved maximum benefit when analysis laboratories were closely integrated with public health programs^2^.

Therefore, during the COVID-19 pandemic, Wayne State University (WSU), the Detroit Health Department (DHD), Henry Ford Health (HFH), the Wayne Health Mobile Unit (WHMU), and the Michigan Department of Health and Human Services (MDHHS) collaborated to establish a genomic surveillance pipeline for SARS-CoV-2 in Southeast Michigan populations. Existing infrastructure, including renovated facilities and well-trained personnel, provided a high-throughput capacity to build upon. Additionally, we established a Center for Emerging Infections with a mission to combat future pandemics and emerging infections in Detroit. Thus, we were well positioned to support public health needs through collaboration with the MDHHS and the CDC. In this report, we described the establishment of this pipeline and highlighted this collaboration as a model demonstrating the importance of building local capacity for detecting SARS-CoV-2 variants and exploring spatiotemporal patterns.

## Materials and methods

### Establishment of research protocols

The Southeast Michigan Genomic Surveillance program operated in accordance with all relevant guidelines and regulations, including the Declaration of Helsinki^8^ and applicable institutional and national research regulations. Experimental protocols were reviewed and approved by the appropriate institutional committees (Wayne State University Institutional Review Board, IRB approval no. IRB20042151; Wayne State University Institutional Biosafety Committee, IBC approval no. IBC20042127). Material Transfer Agreements (MTAs) were executed with Henry Ford Health, the Detroit Health Department, and Ion Diagnostics Laboratories (IDL), which archived samples collected from the Wayne Health Mobile Unit throughout the pandemic period. Written informed consent was obtained from all study participants (or from their legal guardians where applicable) prior to sample collection and participation. All samples received by the Wayne State University laboratory were de-identified.

### Support from the MDHHS

The Michigan Sequencing Academic Partnership for Public Health Innovation and Response (MI-SAPPHIRE) program was initiated by the MDHHS through a CDC Epidemiology and Laboratory Capacity grant in January of 2022. This program recognized the need for genomic surveillance in Michigan and partnered with four universities, including the University of Michigan, Michigan State University, Wayne State University, and Michigan Technological University. The goal was to identify and monitor SARS-CoV-2 variants by expanding the capacity of sequencing and analysis in each university, and ensure sampling covered diverse geographic areas across the state.

### Establishment of the genomic surveillance pipeline

We created a program workflow that underwent numerous iterative attempts, which were systematically evaluated through benchmark exercises to optimize efficiency while upholding quality control standards. The flow included:

#### Sample collection

We aimed for genome sampling reflective of the geographic area targeted and partnered with suppliers to maximize coverage. We transferred all samples to the WSU Integrated Research Laboratories (WIRL) located at the Integrative Biological Sciences (IBio) Center. The system established enabled the pairing of genomic and epidemiologic data in compliance with HIPPA regulations through FORTE OnCore Clinical Trial Data Management System to ensure patient privacy. SARS-CoV-2 samples were received from the following sources:

*Henry Ford Hospital (HFH) samples:* Both retrospective and prospective SARS-CoV-2 samples were collected and therefore sample collection dates ranged from March 2020- November 2023. Nasal swab samples were collected as part of clinical work-up and tested for SARS-CoV-2 by real-time reverse transcription PCR (RT-qPCR) in Microbiology/Serology lab, a division of Pathology and Laboratory Medicine (PALM) that provides various clinical and anatomical pathology lab services for Henry Ford Health. RT-PCR was performed using several SARS-CoV-2 RT-PCR kits on various platforms, including Xpert Xpress SARS-CoV-2 RT-PCR kit for positivity testing on the GeneXpert System. Positive SARS-CoV-2 samples were retrieved by the Infectious Disease Research lab for processing. When available, samples with cycle threshold (Ct) values < 30 were selected and distributed to WSU.

*Wayne Health Mobile Unit (WHMU) and Ion Diagnostic Laboratory (IDL) samples:* In partnership with WHMU, we established a fleet of eight fully upfitted mobile health units (MHUs) which operate 6–7 days a week with a total staff of more than 60 full and part time employees. WMHU serves as the medical service provider on the MHUs, offering health care screenings in the field and meeting the community where they were. These MHUs were deployed to nursing homes, senior living facilities, churches, jails, homeless shelters, and other community-based organizations statewide, with more than 92,000 collective encounters to date. Services offered include, COVID-19 vaccine administration, diagnostic lab-based testing for SARS-CoV-2 by IDL, and other health conditions, point-of-care screenings, vital sign measurements, and linkage to follow-up care and community resources. The MHUs were equipped with medical grade refrigeration and were able to handle sample processing and storage. Clinical procedures were performed by registered nurses and medical assistants, and each medical encounter was documented in the Wayne Health EMR, Athena clinicals®. Through this EMR, MHU staff placed lab orders and scheduled follow-up appointments while in the field. Community Health Workers also were on-site to link patients to social services. All results were communicated with the patients through the Wayne Health Athenahealth Patient Portal. Positive SARS-CoV-2 samples were collected by IDL for processing. Samples were assigned a unique sequence ID and placed in prelabeled tubes provided by IBio.

*Detroit Health Department (DHD) samples:* DHD testing operations focused on essential city workers (police, fire, city staff), outreach to vulnerable populations (skilled nursing centers, homeless shelters), community outbreaks (schools; behavioral health, retail and manufacturing facilities; great-lakes shipping vessels) and community walk-in testing. Samples were collected by nasal swab and processed on Abbott ID-NOW isothermal amplification devices in health department testing clinics. Eluants from positive samples were stored at − 20 °C, then thawed and heat inactivated for 20 min at 60 °C before transport to WSU for processing.

#### Biobanking

The WSU Pathogen Bank, which is a component of the broader WSU Biobank, serves as a repository for specimens collected throughout the study collection basin. These specimens are meticulously categorized and stored. The Oncore Biospecimen Management (BSM) program, a comprehensive software for clinical trials data collection, is utilized for barcoding and categorizing the specimens. This program generates unique barcode labels and captures detailed information about each biospecimen, including its description, acquisition and preparation procedures, and storage location.

To ensure the integrity and security of the specimens, the biobank is housed in a state-of-the-art, temperature-controlled building with keycard-restricted access. The facility is equipped with backup systems to prevent sample loss in the event of a power failure, including emergency power and a backup generator. The ElPro temperature system is used to monitor the freezers, maintaining a consistent temperature of − 80 degrees Celsius. This temperature is strictly monitored, logged, and tracked, with adherence to rigorous biosafety protocols to safeguard the samples and personnel. All samples including original swab and transport solution and nucleic acid isolations are stored at − 80.

WSU expanded its storage capacity and secured dedicated personnel to coordinate the storage, maintenance, and distribution of the samples. All samples were bar-coded and stored, with the associated clinical, demographic, and diagnostic data archived into the existing OnCore system. This required establishing protocols, a data registry system, and monitored − 80 °C refrigerators.

#### Data capture and sample processing

*Sample pre-processing:* At each collaborative clinic, samples were de-identified and assigned a unique sequence ID provided by IBio on prelabeled tubes to maintain patient anonymity. At the IBio research lab, the tubes containing samples were incubated in water bath at 56 °C for 30 min for heat inactivation, and 1 ml of each sample was aliquoted into its respective prelabelled tube in the Biosafety Level-2 (BSL-2) cabinet. Aliquoted samples were stored in sequential order in − 80 °C freezer until transfer for sequencing.

*RNA extraction and RT-qPCR testing:* To standardize the procedure, all samples we received were uniformly tested for SARS-CoV-2 positivity. RNA was extracted from samples using MagMAX Viral/Pathogen Nucleic Acid Isolation Kit (Applied Biosystems, Cat # A48310) using Thermo Scientific’s Kingfisher Apex platform (protocol “MagMax_MVP_Standard_v1”). Briefly, 200 µL of sample were mixed with proprietary DNA/RNA binding magnetic beads. Beads were then washed 1 × with proprietary wash buffer, followed by 2 × washes with 80% Ethanol (Sigma-Aldrich, Cat# E7023-1L) prepared with UltraPure Distilled Water (Invitrogen, Cat# 10977-015). Samples were then air-dried and eluted in 100 µL of proprietary elution solution. Samples were either briefly stored at 4°C for immediate downstream use (RT-qPCR and/or sequencing) or stored at − 80 °C for long term storage. After extraction, RT-qPCR was performed using TaqPath COVID-19 RNase P Combo Kit 2.0 (Applied Biosystems, Cat# A51333) on Thermo Fisher Scientific’s Quant Studio 7 Flex platform according to manufacturer’s protocol. Briefly, 14 µL of purified samples were mixed with 6 µL of reaction mix containing primer/probes that target ORF1a, ORF1b, N Gene regions of SARS-CoV-2 virus and human RNase P as a control. RT-qPCR was performed using the Emergency Use Authorization protocol that can be found on Thermo Fisher’s website.

#### Sequencing and variant assignment

We worked to match the scalability of the project by improving its sequencing capacity by automation. Sequencing libraries and high-throughput sequencing were performed in the Genomic Sciences Core Lab at the IBio Center. SARS-CoV-2 variants were determined using whole genome sequencing with the QIAseq DIRECT Kit. In brief, 5 µL of RNA input was prepared following the “Below 32 Ct protocol” with QIAseq Enhancer and QIAseq Booster A add-ons. Each batch included a positive (AcroMetrix COVID-19 RNA Control (p/n: 954519)) and negative control (QIAGEN Nuclease-free water). Libraries were quantified using Agilent TapeStation 4200 before pooling and sequencing on a NovaSeq 6000 (150 bp × 2; 1% PhiX). After demultiplexing to individual samples, reads were trimmed before aligning to SARS-CoV-2 reference (NC045512)^9,10^. Variants and consensus sequences were determined following the SAMtools pipeline^11–13^. Strains were called using Nextclade and Pangolin^14,15^.

Turnaround times varied depending on accumulation of samples and delivery to WSU. Once received at WSU, samples required 1–2 days for preparation and transfer to the sequencing. 4 plates of 96 were sequenced Friday, and the sequencing was demultiplexed the following Monday. Processing the variant calls took an additional 3 days. Turnaround times decreased as the process became more efficient, averaging 14 days between sample collection and sequencing results.

#### Data management, analysis and sharing

We established a high-performance computing grid for genomic data storage, sharing, and analysis. Bioinformatics analysis of sequencing results encompassed variant, clade, and lineage assignment as well as pathogen identification highlighting the genomic characteristics specific to Southeast Michigan.

Only HFH samples were used in the illustrative analysis of a hospital-based cohort presented:

*Measures:* The COVID-19 case prevalence rate was calculated from numerators of COVID-19 cases divided by denominators of US Census Bureau’s American Community Survey (ACS), 2015–2019. ACS population denominators were based on ZIP code tabulation area (ZCTA) specific estimates for ZIP codes observed in the sample. The mortality rate was calculated from numerators of COVID-19 deaths divided by ACS, 2015–2019, population denominators. The case fatality rate (CFR) was calculated from numerators of deaths divided by cases of COVID-19. Rates use ZCTA- and demographic- (e.g., age, biological sex) specific ACS estimates when possible.

The Robert Graham Center’s ZIP code-level social deprivation index (2019) was used as an area-level measure of social vulnerability. The social deprivation index (SDI) is a composite measure based on percentages of poverty, less than 12 years education, single parent households, renting, overcrowding, households without a car, and unemployed adults under the age 65 years. We created a composite measure of SDI tertiles and participant race to compare social vulnerability in terms of low/medium versus high SDI and Black versus non-Black individuals. We discretized SDI into tertiles to avoid assuming a linear relationship between SDI and COVID-19 mortality. Low/medium SDI collapsed and compared to high SDI as to improve interpretability and preserve power, given the additional stratification for Black race. Further, Black race was incorporated into the measurement given SDI’s composite measure does not include race and given the racial disparities (pertaining to the COVID-19 pandemic) observed within our sample and Michigan overall.

*Statistical analysis:* Descriptive statistics, including counts and proportions, were used to estimate case and mortality rates. Statistical significance was evaluated by non-overlapping 95% confidence intervals (CI) calculated using the Altman^16^ method [RStudio package ‘epiR’ version 2.0.36]. Loess locally weighted regression was utilized to visualize weekly time profiles. Logistic regression with a ZIP code random intercept was used to estimate odds ratios (OR) and 95% CIs between COVID-19 mortality and the composite measure of SDI and Black race. Analyses were conducted in R version 4.3.3 (Posit Software); maps were created using ArcGIS Pro 3.2.0 (Esri Inc.).

*Data sharing:* Conclusions pertinent to decision-makers were shared for public policies and interventions to be drafted. Each of the collaborative clinical partners (HFH, DHD, and WHMU-IDL) directly shared the results with the government agencies.

Additional methodological details can be found in the Supplementary Information.

## Results

### Establishment of genomic surveillance pipeline

**Fig. 1:**
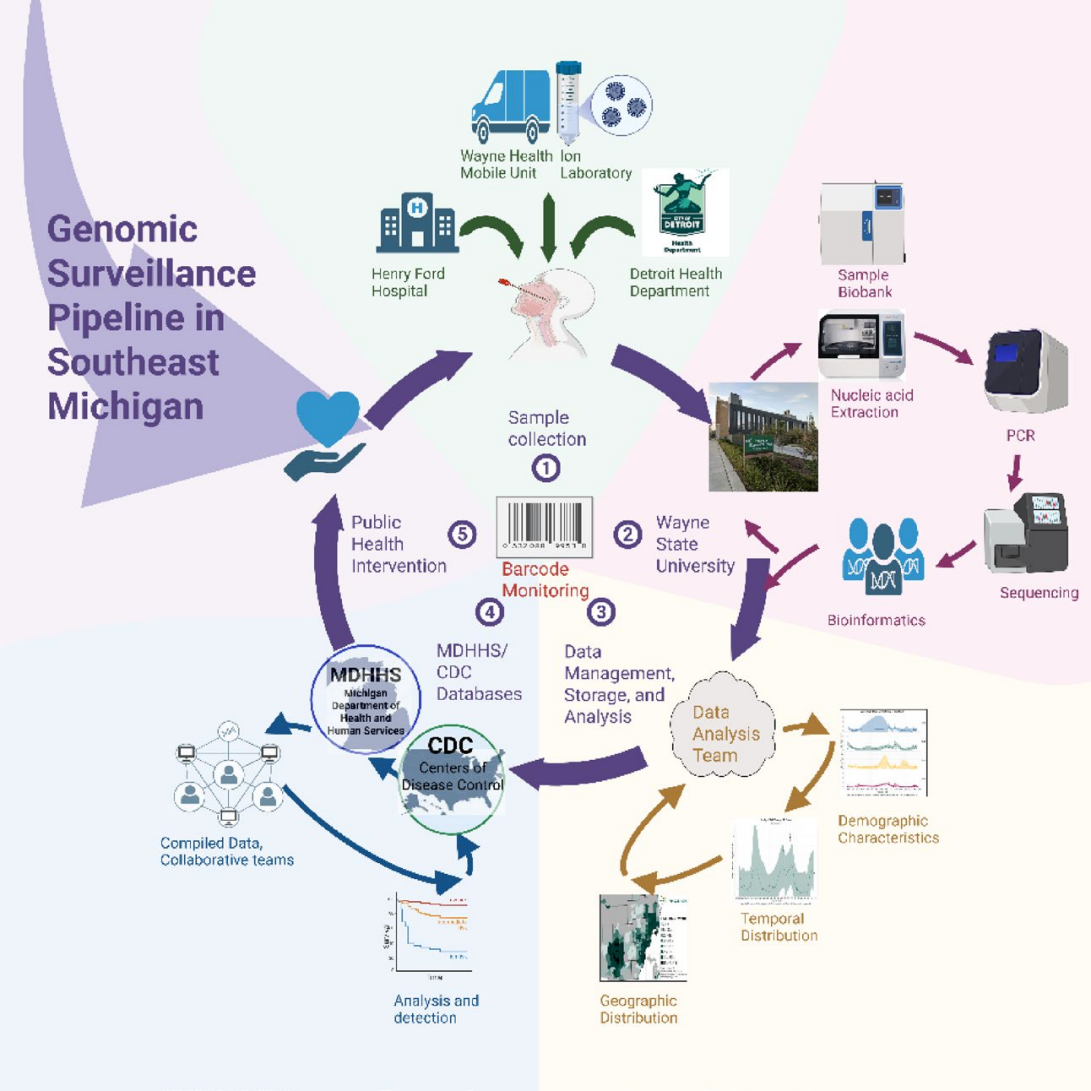
Overview of genomic surveillance workflow. Illustration created by Rola Raychouni using BioRender.com. Reproduced with permission under CC BY 4.0.

We implemented a genomic surveillance program to address a critical gap in Detroit’s public health outbreak tracking system. We achieved the objective of establishing a comprehensive monitoring system capable of both real-time and retrospective analysis of sample sequencing data, aimed at mitigating outbreaks and enhancing vigilance. This initiative not only created an operational technical workflow for sample sequencing but also established the groundwork for robust data mapping infrastructure and opened communication channels that linked the sampling, testing procedures, analytical processes, and subsequent response mechanisms (Fig. 1).

*Sample collection:* We devised a comprehensive surveillance strategy emphasizing targeted sampling representative of Southeast Michigan geographic regions and inclusive of diverse demographic cohorts, spanning age, gender, ethnicity, and socioeconomic status. The aim was to detect signals indicative of transmission hotspots and the emergence of concerning variants, thereby pinpointing populations at heightened risk of infection or severe illness. The sampling approach prioritized regularity and consistency to bring to light trends in transmission dynamics effectively. To achieve these objectives, we collaborated with HFH for hospital-based sampling of patients seeking medical care, DHD for specific populations such as police and other government employees, and WMHU to access various community settings. This combined approach facilitated focused sample collection and rapid response capabilities, particularly in underserved communities with limited healthcare access. The mobile health units played a pivotal role in targeting vulnerable populations such as the elderly, homeless individuals, and those in correctional facilities, where disease transmission rates may be elevated. This approach aimed to improve containment efforts in high-risk environments. WMHU enabled the swift deployment of resources to emerging outbreak areas or evolving epidemiological hotspots, ensuring a quick response to dynamic public health needs.

*Technical workflow:* We built a barcode monitoring system integrated with Laboratory Information Management System (LIMS) facilitating data transfer across all stages of the laboratory workflow. The LIMS platform facilitated inventory management, experiment planning and scheduling, data organization and storage, alongside reporting and analytics capabilities. With LIMS, the entirety of laboratory processes could be effectively tracked, managed, and summarized. The barcode monitoring system tracked samples across diverse stages of the technical workflow, spanning collection, storage, processing, analysis, and data interpretation.

*Analytical processes:* The final stages of the genomic surveillance program involved the data analysis team, tasked with synthesizing data generated through technical workflow and bioinformatics analyses. This team generated visual representations to depict demographic characteristics, temporal trends, and spatial distributions of samples and their sequenced variants. Employing statistical analyses, they assessed the data to validate observed patterns and conclusions, ensuring reliability and significance.

*Response mechanisms:* MDHHS conducted its own analyses and visualizations for public dissemination on their website, which was also linked to the CDC. Open communication channels facilitated collaborative policymaking and intervention efforts throughout the program.

*Public Health intervention:* Once patterns were identified and interventions determined, this iterative process circles back to target communities in need, perpetuating ongoing sampling, testing, and monitoring initiatives (Fig. 1).

### Performance and validation of the program

Throughout the entire genomic surveillance program (Jan 2022–Jul 2024), a total of 7508 samples were collected, barcoded, and archived using the established program. By the time of this report, a total of 6235 samples were RT-qPCR-tested and sequenced. 83.0% of samples underwent sequencing (Fig. S1). Variations in sequencing rates among collection sites stemmed from differences in sample supply logistics and timing from the source. Notably, HFH consistently provided samples (*N* = 4637) in larger quantities and on a regular basis, facilitating their prioritization for processing and resulted in 94.0% of their samples being sequenced. Since these samples were accompanied by comprehensive demographic data, we used them as an example cohort for further spatial, temporal, and characteristics analyses.

### Spatial distribution of collected samples

**Fig. 2:**
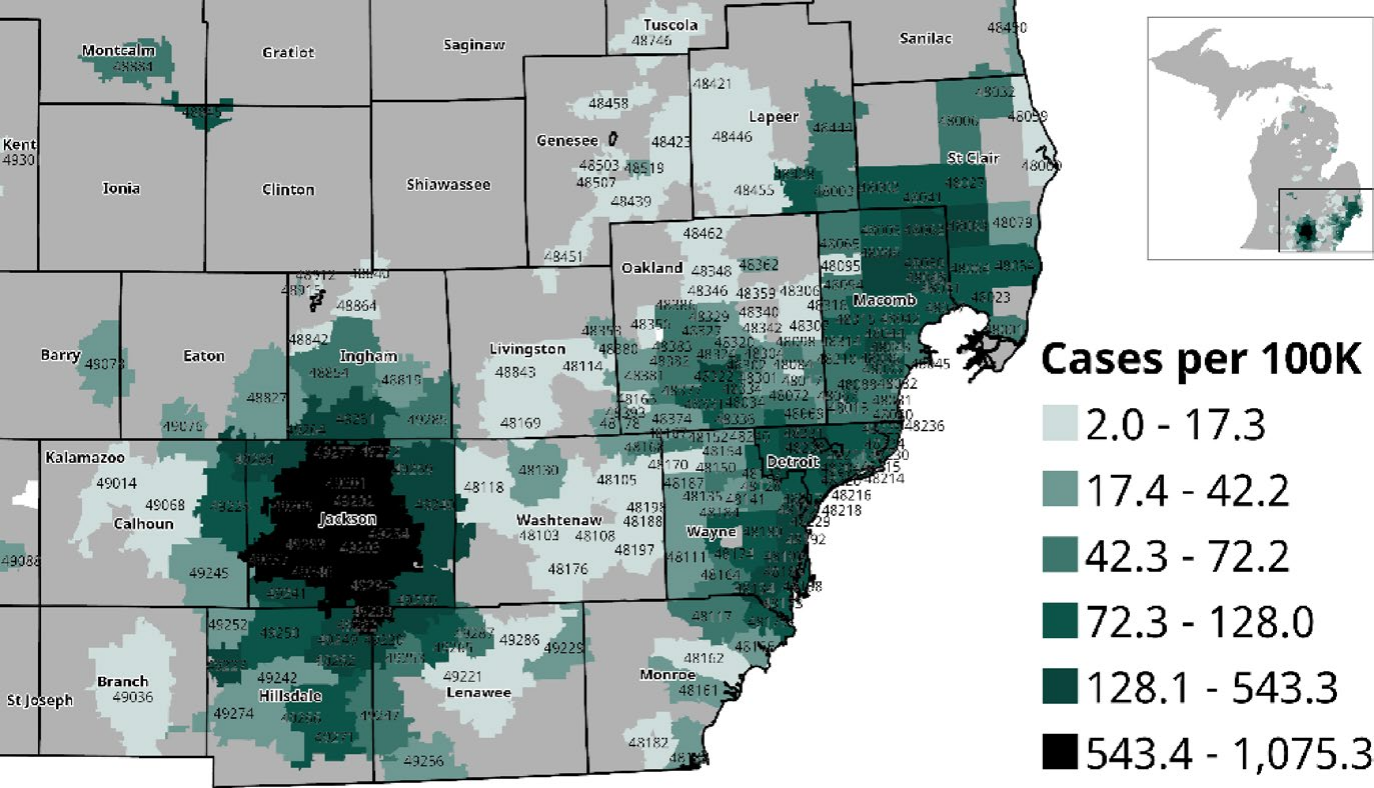
COVID-19 Incidence by ZIP Code. Choropleth map displaying COVID-19 cases per 100,000 population by ZIP code, calculated using the ACS 2015–2019 population denominator. *Note* COVID-19 cases *N* = 4583. Population denominator from American Community Survey 2015–2019; ZCTA-specific totals of 295 Michigan ZIP codes (*N* = 5,728,013 residents).

We mapped HFH samples based on geographic distribution in Southeast Michigan (Fig. 2). Cases were spread across 295 (out of the 989) Michigan ZIP codes primarily in the Southeast region of the lower peninsula, totaling 5,738,013 residents per the Census Bureau’s American Community Survey. Of the 4637 HFH samples, 875 were obtained from hospitalized patients, while 3,762 were collected from individuals in various HFH testing sites. The greatest concentration of cases and deaths were observed in the Jackson County region (Fig. S2). Case fatality rates (CFR) do not follow the same trend, as Genesee County ZIP codes had the highest CFR compared to that of other ZIP codes observed in the samples (Fig. S3).

### Demographic characteristics of patient population and outcome measures

We analyzed the demographic, clinical, and geospatial distribution data of HFH samples. Of the 4637 COVID-19 samples collected, 4583 (98.8%) included a valid Michigan ZIP code and were retained in the analytic sample (Fig. S4). Cases were predominantly ages 20–49 years old [33%, 95% CI (32, 34)], female [57%, 95% CI (56, 59)], and White [55%, 95% CI (54, 57)] (Table S1). Of these, 63.3% were vaccinated [*N* = 2935; 95% CI (62, 65)], with 43% of them boosted [*N* = 1,954; 95% CI (41–44)]. About 19% required hospitalization within 14 days of infection [*N* = 864; 95% CI (18, 20)], and 3.6% died [*N* = 166; 95% CI (3.1, 4.2)] (Table S2).

Overall, the case prevalence rate of SARS-CoV-2 infection was 8.0 cases per 10,000 individuals [*N* = 4583; 95% CI (7.8, 8.2)]. Observed COVID-19 mortality rate was 2.9 deaths per 10,000 individuals [*N* = 166, 95% CI (2.5, 3.4)]. However, it should be noted that these rates should be interpreted with caution since the numerators are not necessarily based on the entire population. CFR was 3.6% [95% CI (3.1, 4.2)] among the samples (Table 1).

**Table 1:** COVID-19 case, mortality, and case fatality rates by demographic characteristics.

	Cases			Deaths			Case fatality	
Characteristics	*N* = 4583	Rate per 10 K	95% CI	*N* = 166	Rate per 10 K	95% CI	%	95% CI
Overall	4583	8.0	(7.77, 8.23)	166	2.90	(2.50, 3.40)	3.62	(3.12, 4.20)
Age group								
< 20	569	4.05	(4.05, 4.07)	1	0.01	(0.01, 0.03)	0.18	(0.03, 0.99)
20–49	1,532	6.97	(6.97, 6.99)	9	0.04	(0.04, 0.06)	0.59	(0.31, 1.11)
50–64	1,122	9.38	(9.38, 9.41)	30	0.25	(0.25, 0.28)	2.67	(1.83, 3.79)
65–74	711	13.09	(13.08, 13.14)	44	0.81	(0.81, 0.87)	6.19	(4.64, 8.21)
75+	649	16.76	(15.52, 18.10)	82	2.12	(2.11, 2.20)	12.63	(10.3, 15.41)
Biological sex								
Female	2,631	8.97	(8.63, 9.31)	73	0.25	(0.20, 0.31)	2.77	(2.21, 3.47)
Male	1,952	6.99	(6.68, 7.30)	93	0.33	(0.27, 0.41)	4.76	(3.90, 5.80)
Race								
White	2,541	9.1	(8.75, 9.46)	99	0.24	(0.20, 0.29)	3.90	(3.21, 4.72)
Black	1,374	12.41	(11.77, 13.08)	55	0.50	(0.38, 0.65)	4.00	(3.09, 5.17)
Other	199	4.12	(3.59, 4.74)	2	0.04	(0.01, 0.15)	1.01	(0.28, 3.59)
Unknown								
Hispanic Ethnicity	189	71.5	(6.20, 8.25)	4	0.15	(0.06, 0.39)	2.12	(0.83, 5.31)

*Case and mortality rates are calculated using American Community Survey (2015–2019) population denominators for observed ZIP codes in the sample (*N* = 5,728,013 individuals). For each demographic variable we retained ZCTA-specific estimates for ZIP codes observed in COVID-19 sequencing data. Case fatality rates are calculated by dividing numerators of total deaths divided by denominators of total cases. 95% confidence intervals (CI) using Wilson Score method.

The prevalence of infection within this population increased with age, with non-overlapping CIs ranging from 4.05 per 10,000 individuals [95% CI (4.05, 4.07)] among those age < 20 to 16.76 per 10,000 [95% CI (15.52, 18.10)] among those 75 years and older, which may have reflected the nature of this hospital-based population. Sex-specific prevalence rates indicate a statistically significant female excess [female: 8.97 per 10 K, 95% CI (8.63, 9.31); male: 6.99 per 10 K, 95% CI (6.68, 7.30)]. However, sex-specific mortality rates suggested a male excess, though not statistically significant [female: 0.25 per 10,000 persons, 95% CI (0.20, 0.31); male: 0.33 per 10,000 persons, 95% CI (0.27, 0.41)] (Table 1).

As for the SARS-CoV-2 variants observed in our samples, Omicron was the most prevalent (*N* = 2,942), accounting for 64% of cases with a rate of 5.1 cases per 10 K individuals [95% CI: (4.95, 5.32)] (Table S3). Mortality was highest among cases carrying variants 20A (European 2 lineage: EU2) [0.12 per 10 K, 95% CI (0.10, 0.15)] and Omicron [0.1 per 10 K, 95% CI (0.07, 0.13)]. Case fatality rate was greatest among variants 19 A + B [7.69%, 95% CI (5.03, 11.58)] and 20A (EU2) [9.65%, 95% CI (7.72, 11.99)], although overlapping CIs indicate no statistical difference (Table 2).

**Table 2:** Relationship between COVID-19 mortality and social deprivation index with black race among COVID-19 cases.

		Model 1		Model 2		Model 3		Model 4	
Covariables		OR	95% CI	OR	95% CI	OR	95% CI	OR	95% CI
Exposure status									
High SDI	Black								
–	–	Ref	–	Ref	–	Ref	–	Ref	–
+	–	0.78	(0.53, 1.15)	0.79	(0.53, 1.16)	0.83	(0.56, 1.23)	0.89	(0.59, 1.33)
–	+	*0.32	(0.08, 0.86)	*0.33	(0.08, 0.90)	0.40	(0.10, 1.10)	*0.33	(0.08, 0.94)
+	+	1.21	(0.84, 1.75)	1.25	(0.86, 1.81)	1.33	(0.91, 1.94)	1.35	(0.91, 1.99)
Biological sex									
Male				Ref	–	Ref	–	Ref	–
Female				*0.57	(0.42, 0.78)	*0.62	(0.45, 0.85)	*0.67	(0.48, 0.92)
Age									
< 65 years						Ref	–	Ref	–
≥ 65 years						*7.41	(5.21, 10.77)	*8.11	(5.66, 11.86)
Variant									
20A								Ref	–
19 A + B								0.71	(0.40, 1.21)
Alpha								0.39	(0.02, 2.01)
Delta								*0.06	(0.004, 0.29)
Omicron								*0.16	(0.11, 0.23)
Unknown/Other								*0.34	(0.19, 0.59)
Observations		4583		4583		4583		4583	
Log Likelihood		− 708.83		− 702.61		− 631.29		− 579.21	
Akaike Inf. Crit		1,425.67		1,415.22		1,274.58		1,180.43	

Complete sample *N* = 4583 COVID-19 cases. Logistic regression model with ZIP code random intercept. Model 1: crude, Model 2: adjusted for biological sex, Model 3: adjusted for biological sex, age ≥ 65 years, Model 4: adjusted for biological sex, age ≥ 65 years, variant. Social Deprivation Index is a composite measure based on percentages of poverty, less than 12 years education, single parent households, renting, overcrowding, households without a car, and unemployed adults under the age 65 years (Robert Graham Center, 2019) and COVID-19 mortality.

*Statistically significant.

### Disparities in virus infection, mortality, and case fatality rate (CFR)

In the HFH cohort, 55% were White, 30% Black, and about 4% were of Hispanic ethnicity. The prevalence rate of infection among Black individuals [12.41 per 10 K, 95% CI (11.77, 13.08)] was approximately 1.4-fold higher than that among White individuals [9.1 per 10 K, 95% CI (8.75, 9.46)]. Mortality rates for Black individuals [0.50 per 10 K, 95% CI (0.38–0.65)] were more than twice those for White individuals (0.24 per 10 K, 95% CI (0.20–0.29)], while CFR were comparable among the two races (Table 1). To further investigate these results, we analyzed the relationship between COVID-19 mortality and social deprivation index (SDI) with Black race among COVID-19 cases in (Table 2). Social Vulnerability appeared to be a major contributor to increased COVID-19 mortality, not race. Among all COVID-19 cases, crude estimates indicate low/medium SDI and Black race were associated with 68% decreased odds of mortality when compared to low/medium SDI and non-Black race [OR: 0.32, 95% CI (0.08, 0.86)]. This association holds after adjusting for age, biological sex, and variant [OR: 0.33, 95% CI (0.08, 0.94)] (Table 2). We repeated the model among the 864 participants who were admitted to the hospital within 14 days of testing positive to account for the differences in case severity (Table S4). Crude and adjusted estimates follow the same pattern observed in the full sample.

### Temporal distribution of samples

**Fig. 3:**
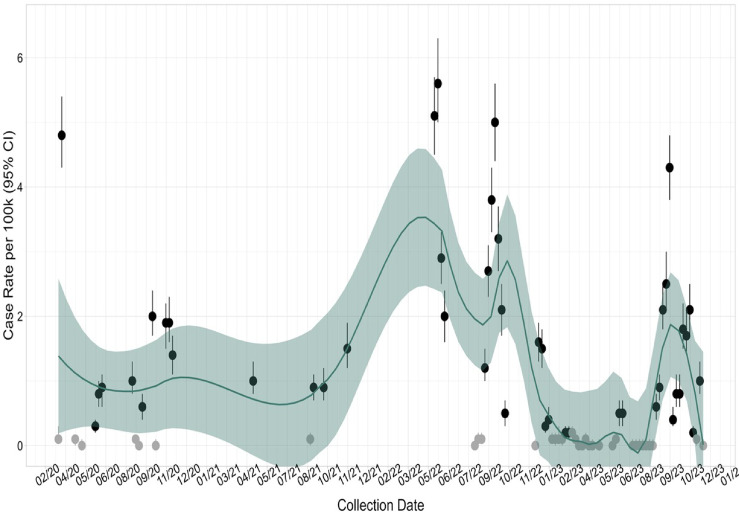
Weekly Incidence per 100,000 with Loess Smoothing. Weekly incidence of COVID-19 per 100,000 population with Loess smoothing (spa* N* = 0.25) applied. *Notes* Grey dots indicate < 10 cases. COVID-19 cases *N* = 4583; denominator *N* = 5,728,013. Population denominator from American Community Survey 2015–2019; ZCTA-specific totals of 295 Michigan ZIP codes. 95% Confidence interval (CI). Loess smoothing paramete* r* = 0.25.

Cases were distributed across 28 out of the 45 months sample collection dates spanned from March 2020 through November 2023. Based on our sample, weekly prevalence rates were the highest during March 2020, May 2022, September 2022, and September 2023 (Fig. 3). Weekly race- and Hispanic-specific COVID-19 rates follow about the same overall trend, with exception to the first weeks where a statistically significant excess among Blacks was observed (Fig. S5).

**Fig. 4:**
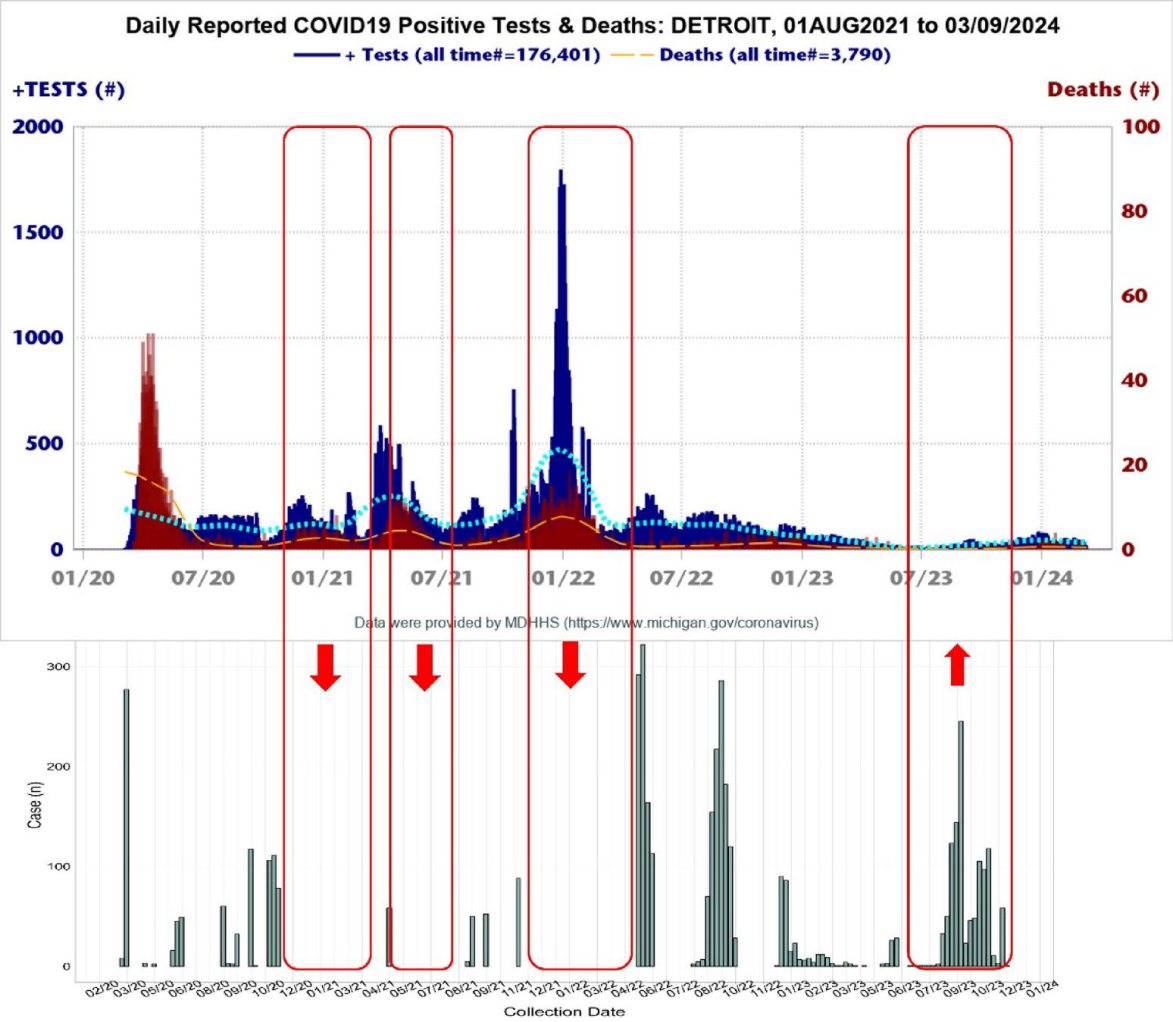
Comparison of weekly cases with the michigan department of health. Comparison of temporal distribution of COVID-19 cases between the Michigan department of health and our sample set. The graphs illustrate discrepancies in COVID-19 case distribution throughout the pandemic.

In comparing the temporal distribution of COVID-19 caseloads between our sample set and data from the MDHHS^17^, we observed notable discrepancies. Specifically, gaps in our sampling corresponded to spikes in the state’s surveillance data during January 2021, June 2021, and January 2022. Conversely, we detected a surge in cases in July 2023, a period during which MDHHS reported near-zero cases (Fig. 4).

### Distribution of variants over time

We collapsed variants detected through the program into the following categories: 19 A + B (19A and 19B), 20A (EU2) (20A, 20B, 20C, 20E, 20F, 20G), Alpha (20I), Delta (21A, 21I, 21 J), Epsilon (21C), Mu (21H), and Omicron (21 K, 21L, 21 M). The 20A (EU2) variant dominated through April 2021 when Delta appeared. The Delta wave was short-lived as Omicron dominated throughout the rest of the pandemic (Fig. S6).

**Fig. 5:**
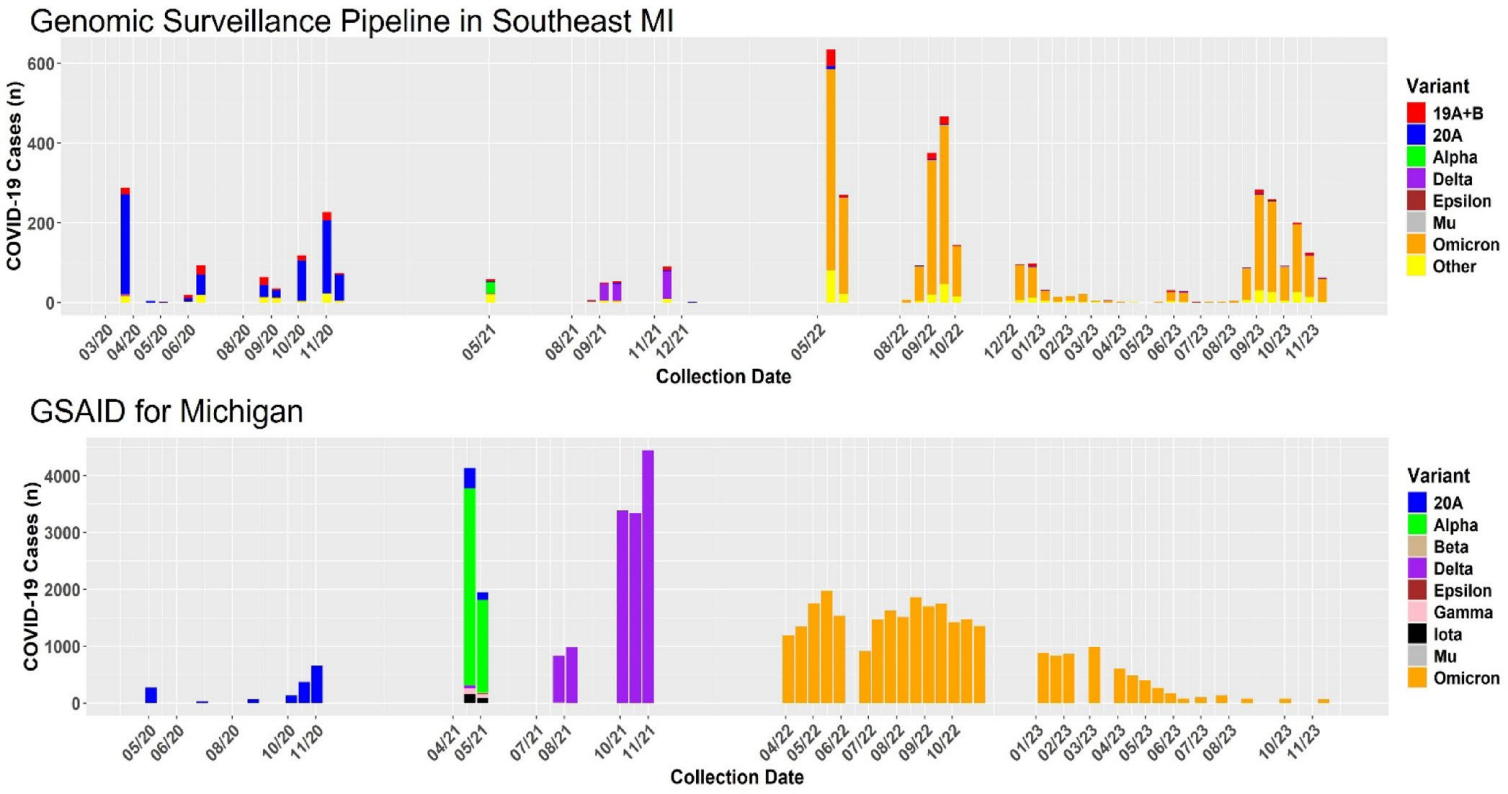
COVID-19 variant trends in program samples against GISAID trends in Michigan from 2020 to 2023. The graphs show the distribution of SARS-CoV-2 variants over time, using the number of sequenced samples (y-axis) against the collection date (x-axis). Michigan samples were retrieved from the GISAID database and represent variant surveillance in Michigan over the same dates HFH samples were collected. Variants such as Alpha, Delta, and Omicron are color-coded with the same colors in both sample sets to highlight their emergence and dominance across different time points.

The variant pattern over time follows the Michigan trend as posted by CoVariants^18^ enabled by Global Initiative on Sharing All Influenza Data (GISAID) database^19^ for the state of Michigan (Fig. 5). A strong positive correlation (*r* = 0.98) was observed (Fig. S7), indicating a close agreement between the variant distributions detected by GISAID and those identified in the program’s sample set.

### Distribution of variants over geography

Among all the variants observed, Omicron has the widest spread across Michigan, reaching into the Upper Peninsula (Fig. S8). However, the highest prevalence rates of Omicron infection were observed in the Detroit Metropolitan Area, including Detroit proper and its surrounding regions (Fig. S9).

### Mutation patterns and exploratory phylogenetic analysis

To ensure consistency and avoid retrospective discrepancies, we used updated Nextclade versions for variant assignments from 2020 to 2023. Consequently, we reconstructed the phylogenetic tree using the most recent Nextclade version (V2.14.0) (Fig. S10). Only samples with good overall quality control status were used to generate the tree and to assess their relationship to circulating variants in Michigan (Fig. S11).

We attempted a statistical analysis, but identifying a period with comparable sampling quantity and distribution was challenging. GISAID exhibited fluctuations in sequence submissions, with periods of high and low activity, while HFH samples were submitted intermittently, primarily during overflow events (Fig. S12). The analysis compared 684 HFH samples with 41,290 complete, high-coverage human SARS-CoV-2 genomes from GISAID^19^ for Michigan using Nextclade, spanning the period between Jan. 2020–Dec.2021, with all duplications removed. Chi-square tests showed significant differences in sample distributions between the HFH and GISAID samples per month (*P* < 0.001) and year (*P* < 0.001). As can be seen, there were many more HFH samples collected in 2020 than in 2021, while the majority of GISAID samples were from 2021. HFH samples had Delta as the major variants, while GISAID samples were dominated by Delta and Alpha strains.

Nextstrain clade distributions also differed significantly between the two datasets (*P* < 0.001), with HFH samples enriched in clades 20C and 20G, whereas GISAID samples were dominated by 21 J. Similarly, Pangolin lineage distributions were significantly different (*P* < 0.001), with HFH samples showing higher proportions of B.1 and B.1.2, while GISAID samples were enriched in B.1.1.7 and AY.103 (Attachment 1). These findings suggest distinct sampling biases and/or epidemiological trends between the HFH and GISAID datasets.

## Discussion

In this study, we aimed to document the successful establishment of the genomic surveillance pipeline for monitoring the SARS-CoV-2 outbreak in the Detroit Metropolitan and Southeast Michigan area. We highlighted an academic–public health partnership that integrated genomic surveillance as a rapid and flexible service with structured result communication and governance to support the local population. We emphasize that this work aims to demonstrate the feasibility and outcomes of the establishment of such a collaborative pipeline rather than scientific discovery of the pandemic.

SARS-CoV-2 presented with a high mutation rate that contributed to its rapid global spread^20–24^. The continuous mutations in the genome, necessitated constant monitoring to stay ahead of the spread. In addition to SARS-CoV-2, Detroit experienced outbreaks of a wide variety of contagious pathogens including influenza, respiratory illnesses, measles, and Mpox. As a result, establishing the genomic surveillance program in Southeast Michigan represented a critical tool to directly address these concerns, provide a complementary means of understanding viral transmission dynamics, and prepare for current and future outbreaks.

The infrastructure required for monitoring viruses in the city of Detroit and neighboring areas was lacking and therefore, we were tasked with creating a program de novo. We built on previous initiatives that outlined objectives necessary for an operational pipeline. The goal was a program whose accuracy, reliability, and scalability were subjected to evaluation through benchmark exercises. Its modular technologies enabled pathogen detection while also observing cost-effective workflows to manage resource requirements efficiently. In addition, establishing data mapping capabilities was imperative to integrate and harmonize data from diverse sources and formats, particularly for integrating epidemiological and genomic data into large-scale systems. The data analysis was to yield conclusions pertinent to decision-makers at various levels, ranging from local to national. Our program was to remain adaptable to potential changes to ensure long-term relevance and impact^25–27^.

We met these objectives by leveraging pre-existing local expertise and infrastructure in viral genomics, strong commitment from HFH, WHMU, and the DHD, as well as collaborative partnerships with public health entities such as the MDHHS and CDC. Our findings offer evidence that an academic-public health partnership can detect SARS-CoV-2 variant circulation (Figs. 5, S7).

### Limitations

The retrospective nature of the analysis done on exclusively HFH samples, limited the findings to patients who sought medical care at HFH and are not representative of the entire region or all COVID-19 cases in the area. This could explain why the data does not align well with case trends in Detroit and shows temporal gaps and spikes at various points (Fig. 4). The uneven temporal distribution of samples limits the ability to draw definitive conclusions or make direct comparisons of estimates of prevalence and associated mortality for variants and races (Figs. S13, S14). Sample collection was intermittent and provided in events of overflow causing sample bias to emerge as a significant limitation to a sound and informative statistical analysis (Fig. S12). In addition, the entire program could be optimized for greater speed and efficiency with turnaround times between sample collection and sequencing reduced.

Sample collection stands as a fundamental and influential step in the effectiveness of the program’s outcomes and subsequent recommendations generated. The validity and impact of the results depend on the comprehensiveness and representativeness of sample collection. Without this critical foundation, subsequent analytical steps may lack reliability, limiting the study’s ability to inform meaningful conclusions and decisions. For instance, Jackson County had the highest case count and mortality rates (Figs. 2, S2), while Genesee County exhibited the highest CFR (Fig. S3). This disparity could result from unequal sampling or reflect Genesee County’s population decline of 5.4% since 2010, with the 65 + age group growing by 30.7% between 2010 and 2022. Given that older adults are at the highest risk for severe outcomes from COVID-19, with over 81% of deaths occurring in individuals aged 65 and older, this demographic shift could explain the observed trend^28,29^. Therefore, comprehensive, inclusive, and extensive sampling is essential, and could be met by widening the data analysis to include samples from the mobile units resulting in a more community-representative picture. This, accompanied by rapid analysis and sharing of results, allows early detection, prevents transmission, and supports the validity of the observed patterns. The establishment of this program represented an important initial step; however, it is evident that further funding, development, and refinement are required. Nevertheless, these limitations are expected to be considered for any future effort in response to similar urgent public health issues.

## Conclusion

This paper outlined the process, challenges, and efforts involved in creating this program, serving as a model for others undertaking similar tasks. While this model is neither perfect nor complete, it is a work in progress and is shared here as a foundation for further refinement by those developing similar systems. The infrastructure established is sustainable and provides a robust framework adaptable for future outbreaks, including RSV, influenza, and Mpox surveillance, pending new funding. Looking forward, we are better positioned to combat the spread of viruses and anticipate outbreaks, thereby preventing the level of unpreparedness experienced during the COVID-19 pandemic.

## Supplementary Information

Below is the link to the electronic supplementary material.

Supplementary Material 1

Supplementary Material 2

## Data Availability

The datasets generated and/or analyzed during the current study are available in the National Library of Medicine repository, BioSample/NCBI (PRJNA1332824).
