# Supplementary material for: Implementation of SARS-CoV-2 genomic surveillance during the COVID-19 pandemic through an academic–public health collaboration in southeast Michigan
Source: Sci Rep. 2026 Feb 24;16:8680. doi: 10.1038/s41598-026-39974-7 (PMC12979760; doi:10.1038/s41598-026-39974-7)
Supplement: Supplementary file 2 — Supplementary Material 2 [file 41598_2026_39974_MOESM2_ESM.docx]

**Implementation of SARS-CoV-2 Genomic Surveillance During the COVID-19 Pandemic Through an Academic–Public Health Collaboration in Southeast Michigan**

Rola Raychouni^1^, Xiangmin Zhang^1^, Samantha J. Bauer^2^, Benjamin Wasinski^3^, Katherine Gurdziel^4,5^, Nivisa Vakeesan^1^, Paige Stanton^1,6^, Anthony T. Lagina III^3^, Michael Mossing^7^, Geehan Suleyman^8^, Jagjeet Kaur^8,^ Maryssa Trupiano^8^, Phillip Levy^3^, Paul E. Kilgore^9,10^, Marcus Zervos^8,10^, Steven Korzeniewski^2^, Wanqing Liu^1,5*^

^1^Department of Pharmaceutical Sciences, Eugene Applebaum College of Pharmacy and Health Sciences, Wayne State University, Detroit, MI 48201

^2^Department of Family Medicine and Public Health Sciences, Wayne State University, Detroit, MI 48201

^3^Department of Emergency Medicine, Wayne State University, Detroit, MI 48201

^4^Genome Sciences Core, Wayne State University, Detroit, MI 48202

^5^Department of Pharmacology, School of Medicine, Wayne State University, Detroit, MI 48201

^6^College of Health Sciences, Purdue University, West Lafayette, IN 47907

^7^Department of Biochemistry, Microbiology, and Immunology, Wayne State University, Detroit, MI 48201

^8^Department of Medicine, Division of Infectious Diseases, Henry Ford Health, Detroit, MI 48202

^9^Department of Pharmacy Practice, Eugene Applebaum College of Pharmacy and Health Sciences, Wayne State University, Detroit, MI 48201

^10^Department of Internal Medicine, Wayne State University 48201

*Corresponding author: Wanqing Liu, PhD. Department of Pharmaceutical Sciences, Eugene Applebaum College of Pharmacy and Health Sciences; Department of Pharmacology, School of Medicine, Wayne State University. Integrative Bioscience Center, 6135 Woodward Ave, Detroit, MI 48202. Email: [wliu@wayne.edu](mailto:wliu@wayne.edu)

This work was supported by the Michigan Department of Health and Human Services [grant: MI-SAPPHIRE project]

**SUPPLEMENTAL METHODS**

**Data management, analysis and sharing**

WSU established a high-performance computing grid for genomic data storage, sharing, and analysis. Bioinformatics analysis of sequencing results encompassed variant, clade, and lineage assignment as well as pathogen identification highlighting the genomic characteristics specific to Southeast Michigan. The genomic surveillance team also collaborated with a data analysis team (S.K) which conducted molecular epidemiological studies tracking the spread and evolution of the virus over time and geographic area. Sequencing, bioinformatics, and demographic data compiled by the data analysis team were analyzed and interpreted. They created the graphs presented in this descriptive paper illustrating the temporal and spatial distribution of viral evolution within the region.

*Data sharing:* Conclusions pertinent to decision-makers are shared for public policies and interventions to be drafted. Each of the collaborative clinical partners (HFH, DHD, and Wayne Health-IDL) directly share the results with the government agencies. The data from master excel sheets used to communicate with WSU are matched with the end of the EPIC charts and the information is uploaded to the state of Michigan’s secure website. The program for this is run in SAS and the sequenced data is matched to the patient’s information and demographics. Their sequenced result is also matched to the SARS-CoV-2 test order in EPIC, and the information for the ordering physician is then extracted. The data is formatted to the specifications from the MDHHS into a .csv file, at which point an upload is attempted. The MDHHS website has encryption, and special access must be granted for the particular area where the data is uploaded. When the file is uploaded, it immediately checks for data quality. If there are any issues flagged, it is rejected and gives the error – one error at a time each upload attempt. They can vary from a null physician address to an illegal character – for example, no “/” characters are allowed in the street address. The personnel investigate the error and make the adjustments in SAS as needed until it passes the validation checker. Once it passes, submission is allowed.

**SUPPLEMENTAL TABLES**

| **Table S1. Description of Sample Characteristics.** | | | | | | | | | | | | | | |  |
| --- | --- | --- | --- | --- | --- | --- | --- | --- | --- | --- | --- | --- | --- | --- | --- |
|  | | | | **Cases (n)** | | | **Sample Proportions** | | | | | | | |  |
| **Characteristics** | | | | N=4,583 | | | % | | | | 95% CI | | | |  |
|  | | | |  | | |  | | | |  | | | |  |
| **Age Group** | | | |  | | |  | | | |  | | | |  |
| <20 | | | | 569 | | | 12.42 | | | | (11.49, 13.40) | | | |  |
| 20-49 | | | | 1,532 | | | 33.43 | | | | (32.08, 34.81) | | | |  |
| 50-64 | | | | 1,122 | | | 24.48 | | | | (23.26, 25.75) | | | |  |
| 65-74 | | | | 711 | | | 15.51 | | | | (14.49, 16.59) | | | |  |
| 75+ | | | | 649 | | | 14.16 | | | | (13.18, 15.20) | | | |  |
|  | | | |  | | |  | | | |  | | | |  |
| **Biological Sex** | | | |  | | |  | | | |  | | | |  |
| Female | | | | 2,631 | | | 57.41 | | | | (55.97, 58.83) | | | |  |
| Male | | | | 1,952 | | | 42.59 | | | | (41.17, 44.03) | | | |  |
|  | | | |  | | |  | | | |  | | | |  |
| **Race** | | | |  | | |  | | | |  | | | |  |
| White | | | | 2,541 | | | 55.44 | | | | (54.00, 56.88) | | | |  |
| Black | | | | 1,374 | | | 29.98 | | | | (28.67, 31.32) | | | |  |
| Other | | | | 199 | | | 4.34 | | | | (3.79, 4.97) | | | |  |
| Unknown/Missing | | | | 469 | | | 10.23 | | | | (9.39, 11.14) | | | |  |
|  | | | |  | | |  | | | |  | | | |  |
| **Hispanic Ethnicity** | | | | 189 | | | 4.12 | | | | (3.59, 4.74) | | | |  |
|  | | | |  | | |  | | | |  | | | |  |
| N=4,583 COVID-19 cases  Sample proportion and 95% confidence intervals (CI) using Wilson Score method. | | | | | | | | | | | | | | |  |
| **Table S2. Description of Sample Outcomes.** | | | | | | | | | | | | | | |  |
|  | | | | | | **Cases (n)** | | | **Sample Proportions** | | | | | |  |
| **Characteristics** | | | | | | N=4,583 | | | % | | | | 95% CI | |  |
|  | | | | | |  | | |  | | | |  | |  |
| **Vaccinated** | | | | | | 2935 | | | 63.28 | | | | (61.88, 64.66) | |  |
|  | | | | | |  | | |  | | | |  | |  |
| **Received Booster** | | | | | | 1,954 | | | 42.64 | | | | (41.21, 44.07) | |  |
|  | | | | | |  | | |  | | | |  | |  |
| **14-Day Post COVID Hospital Admission** | | | | | | 864 | | | 18.85 | | | | (17.75, 20.01) | |  |
|  | | | | | |  | | |  | | | |  | |  |
| **Intensive Care Unit (ICU)** | | | | | | 215 | | | 4.69 | | | | (4.12, 5.34) | |  |
|  | | | | | |  | | |  | | | |  | |  |
| **Deceased** | | | | | | 166 | | | 3.62 | | | | (3.12, 4.20) | |  |
|  | | | | | |  | | |  | | | |  | |  |
| **Discharge Status** | | | | | |  | | |  | | | |  | |  |
| Another Healthcare Facility* | | | | | | 198 | | | 4.32 | | | | (3.77, 4.95) | |  |
| Court/Law Enforcement | | | | | | 2 | | | 0.04 | | | | (0.01, 0.16) | |  |
| Died | | | | | | 77 | | | 1.68 | | | | (1.35, 2.09) | |  |
| Home** | | | | | | 549 | | | 11.98 | | | | (11.07, 12.95) | |  |
| Hospice | | | | | | 24 | | | 0.52 | | | | (0.35, 0.78) | |  |
| Left Against Medical Advice | | | | | | 12 | | | 0.26 | | | | (0.15, 0.46) | |  |
| Unknown/Missing | | | | | | 3,721 | | | 81.19 | | | | (80.03, 82.30) | |  |
|  | | | | | |  | | |  | | | |  | |  |
| N=4,583 COVID-19 cases  Sample proportion and 95% confidence intervals (CI) using Wilson Score method.  *Another healthcare facility includes short term hospitals, behavioral health, psychiatric hospitals, nursing homes, rehabilitation, and long-term care facilities.  **Home includes home healthcare. | | | | | | | | | | | | | | |  |
| **Table S3. COVID-19 Case, Mortality, and Case Fatality Rates Overall and by Variant.** | | | | | | | | | | | | | | |  |
|  | **Cases** | | | | **Mortality** | | | | | | | **Case Fatality** | | | |
| **Variants** | **N=4,583** | **Rate per 10K** | **95% CI** | | **n=166** | | | **Rate per 10K** | | **95% CI** | | **%** | | **95% CI** | |
|  |  |  |  | |  | | |  | |  | |  | |  | |
| **Overall*** | 4,583 | 8.0 | (7.77, 8.24) | | 166 | | | 2.9 | | (2.50, 3.30) | | 3.62 | | (3.12, 4.20) | |
|  |  |  |  | |  | | |  | |  | |  | |  | |
| **Variant** |  |  |  | |  | | |  | |  | |  | |  | |
| **19 A+B** | 260 | 0.45 | (0.40, 0.51) | | 20 | | | 0.03 | | (0.02, 0.05) | | 7.69 | | (5.03, 11.58) | |
| **20A** | 736 | 1.28 | (1.19, 1.38) | | 71 | | | 0.12 | | (0.10, 0.15) | | 9.65 | | (7.72, 11.99) | |
| **Alpha** | 29 | 0.05 | (0.03, 0.07) | | 1 | | | 0.002 | | (0, 0.01) | | 3.45 | | (0.61, 17.18) | |
| **Delta** | 165 | 0.29 | (0.24, 0.33) | | 1 | | | 0.002 | | (0, 0.01) | | 0.61 | | (0.11, 3.35) | |
| **Omicron** | 2,942 | 5.14 | (4.95, 5.32) | | 57 | | | 0.10 | | (0.07, 0.13) | | 1.94 | | (1.45, 2.50) | |
| **Unknown/Other** | 451 | 0.79 | (0.71, 0.86) | | 16 | | | 0.03 | | (0.01, 0.04) | | 3.55 | | (2.20, 5.69) | |
| Note: *Overall case and mortality rates are calculated using American Community Survey (2015-2019) population denominators for observed ZIP codes in the sample (N=5,728,013 individuals). Case fatality rates are calculated by dividing numerators of total deaths divided by denominators of total cases. 95% confidence intervals (CI) using Wilson Score method. | | | | | | | | | | | | | | | |

| **Table S4. Relationship Between COVID-19 Mortality and Social Deprivation Index with Black race among COVID-19 cases admitted to the hospital within 14 days of testing positive.** | | | | | | | | | |
| --- | --- | --- | --- | --- | --- | --- | --- | --- | --- |
|  | | **Model 1** | | **Model 2** | | **Model 3** | | **Model 4** | |
| **Covariables** | | **OR** | **95% CI** | **OR** | **95% CI** | **OR** | **95% CI** | **OR** | **95% CI** |
| **Exposure Status** | |  |  |  |  |  |  |  |  |
| **High SDI** | **Black** |  |  |  |  |  |  |  |  |
| - | - | Ref. | -- | Ref. | -- | Ref. | -- | Ref. | -- |
| + | - | 0.73 | (0.46, 1.16) | 0.74 | (0.46, 1.16) | 0.77 | (0.48, 1.22) | 0.80 | (0.49, 1.27) |
| - | + | *0.15 | (0.02, 0.49) | *0.15 | (0.02, 0.49) | *0.17 | (0.03, 0.59) | *0.14 | (0.02, 0.50) |
| + | + | 0.66 | (0.43, 1.02) | 0.67 | (0.43, 1.02) | 0.71 | (0.46, 1.11) | 0.72 | (0.46, 1.13) |
|  | |  |  |  |  |  |  |  |  |
| **Biological Sex** | |  |  |  |  |  |  |  |  |
| Male | |  |  | Ref. | -- | Ref. | -- | Ref. | -- |
| Female | |  |  | 0.89 | (0.62, 1.29) | 0.88 | (0.61, 1.27) | 0.9 | (0.62, 1.31) |
|  | |  |  |  |  |  |  |  |  |
| **Age** | |  |  |  |  |  |  |  |  |
| <65 years | |  |  |  |  | Ref. | -- | Ref. | -- |
| ≥65 years | |  |  |  |  | *3.28 | (2.17, 5.10) | *3.50 | (2.29, 5.49) |
|  | |  |  |  |  |  |  |  |  |
| **Variant** | |  |  |  |  |  |  |  |  |
| 20A | |  |  |  |  |  |  | Ref. | -- |
| 19 A+B | |  |  |  |  |  |  | 0.86 | (0.44, 1.62) |
| Alpha | |  |  |  |  |  |  | 1.61 | (0.06, 27.60) |
| Delta | |  |  |  |  |  |  | 0.45 | (0.02, 2.54) |
| Omicron | |  |  |  |  |  |  | *0.40 | (0.26, 0.61) |
| Unknown/Other | |  |  |  |  |  |  | *0.51 | (0.25, 0.97) |
|  | |  |  |  |  |  |  |  |  |
| Observations | | 864 |  | 864 |  | 864 |  | 864 |  |
| Log Likelihood | | -379.54 |  | -379.35 |  | -362.23 |  | -352.2 |  |
| Akaike Inf. Crit. | | 767.07 |  | 768.71 |  | 736.46 |  | 726.41 |  |
| Note: Subsample of COVID-19 cases admitted to hospital with 14 days of testing positive (n=864). Logistic regression model with ZIP code random intercept. Model 1: crude, Model 2: adjusted for biological sex, Model 3: adjusted for biological sex, age ≥ 65 years, Model 4: adjusted for biological sex, age ≥ 65 years, variant. Social Deprivation Index is a composite measure based on percentages of poverty, less than 12 years education, single parent households, renting, overcrowding, households without a car, and unemployed adults under the age 65 years (Robert Graham Center, 2019) and COVID-19 mortality. *Statistically significant. | | | | | | | | | |

**SUPPLEMENTAL FIGURES**

**Figure S1:**

**Figure S1: Distribution of Samples by Stage of Genomic Surveillance Pipeline per Source.** Overview of sample count and distribution by stage of genomic surveillance pipeline. Samples were received from sources starting Dec 2022 through Mar 2024.

**Figure S2:**

**
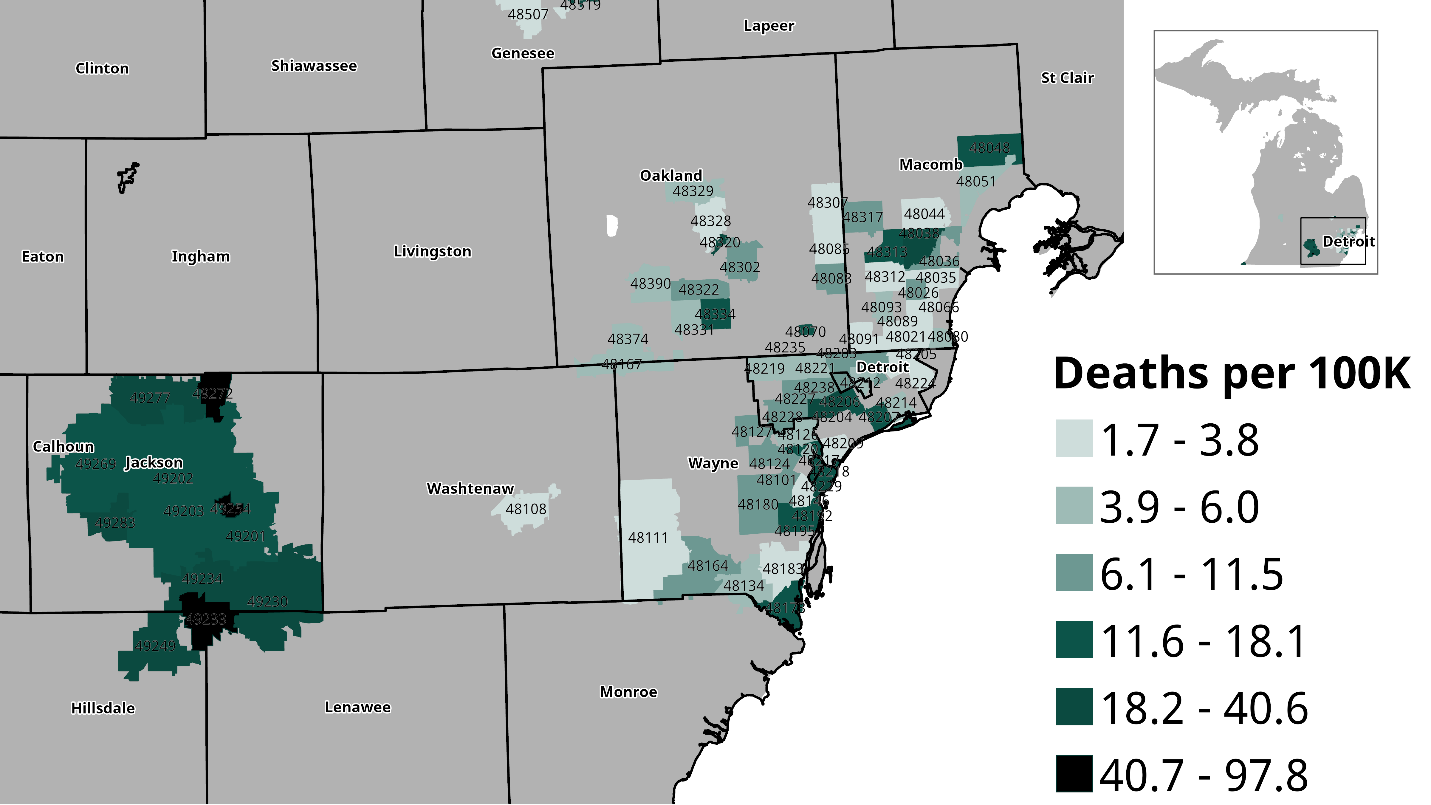
**

**Figure S2. COVID-19 Mortality Rate by ZIP Code.** Choropleth map of COVID-19 mortality rates (deaths per 100,000) by ZIP code. Mortality data include n=166 COVID-19-related deaths

**Figure S3:**


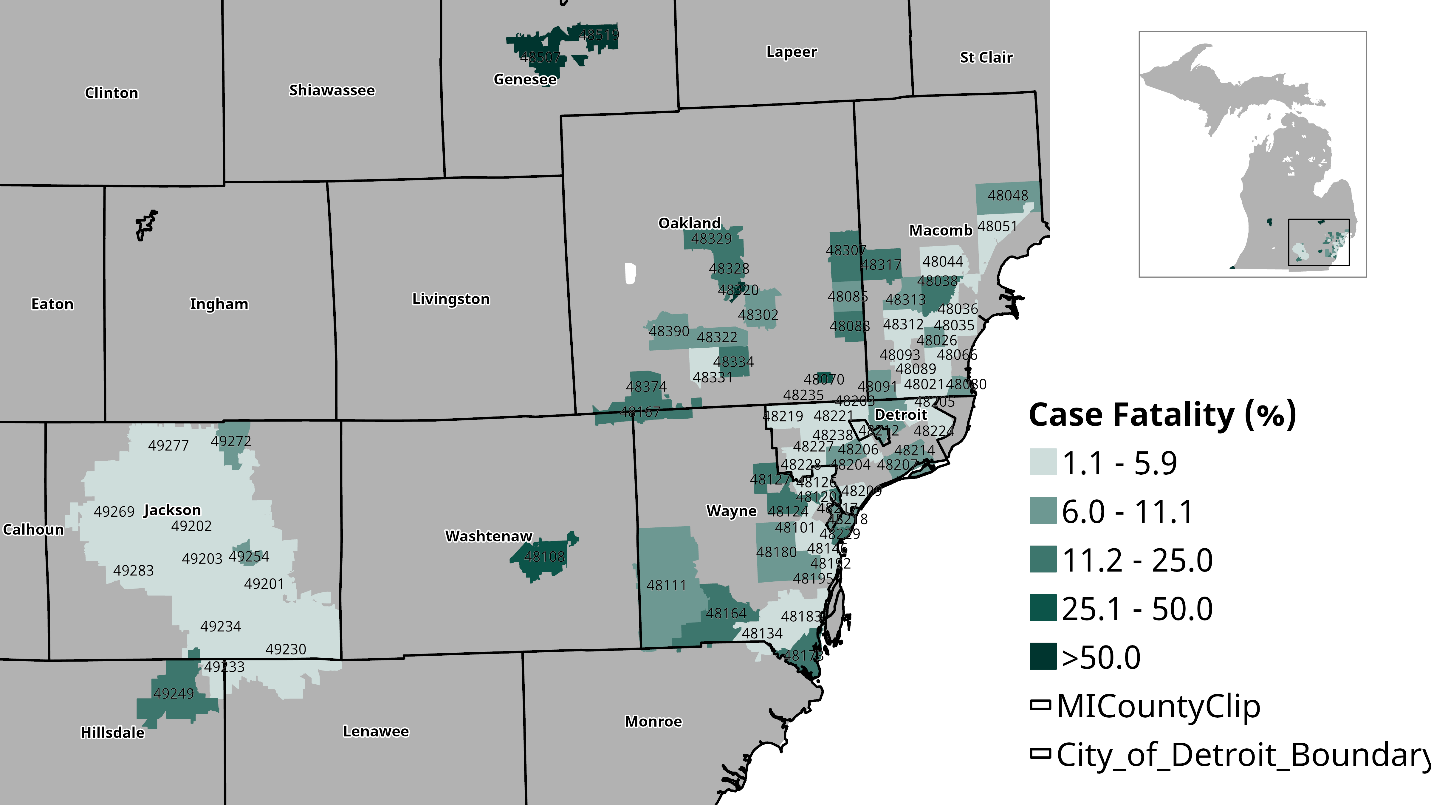


**Figure S3. COVID-19 Case Fatality Rate by ZIP Code.** Choropleth map presenting ZIP code-level case fatality rates, defined as the proportion of deaths among confirmed cases (n=166 deaths, 4,583 cases).

**Figure S4:**

**COVID-19 Cases**

N=4,637

**Michigan Cases**

n=4,583

**Missing ZIP code n=12**

**Invalid/Non-MI ZIP n=42**

**Figure S4. Analytic Sample Consort Diagram.** Flowchart detailing the selection criteria and derivation of the final analytic sample (n=4,583 COVID-19 cases) used in the SAPPHIRE study.

**Figure S5:**


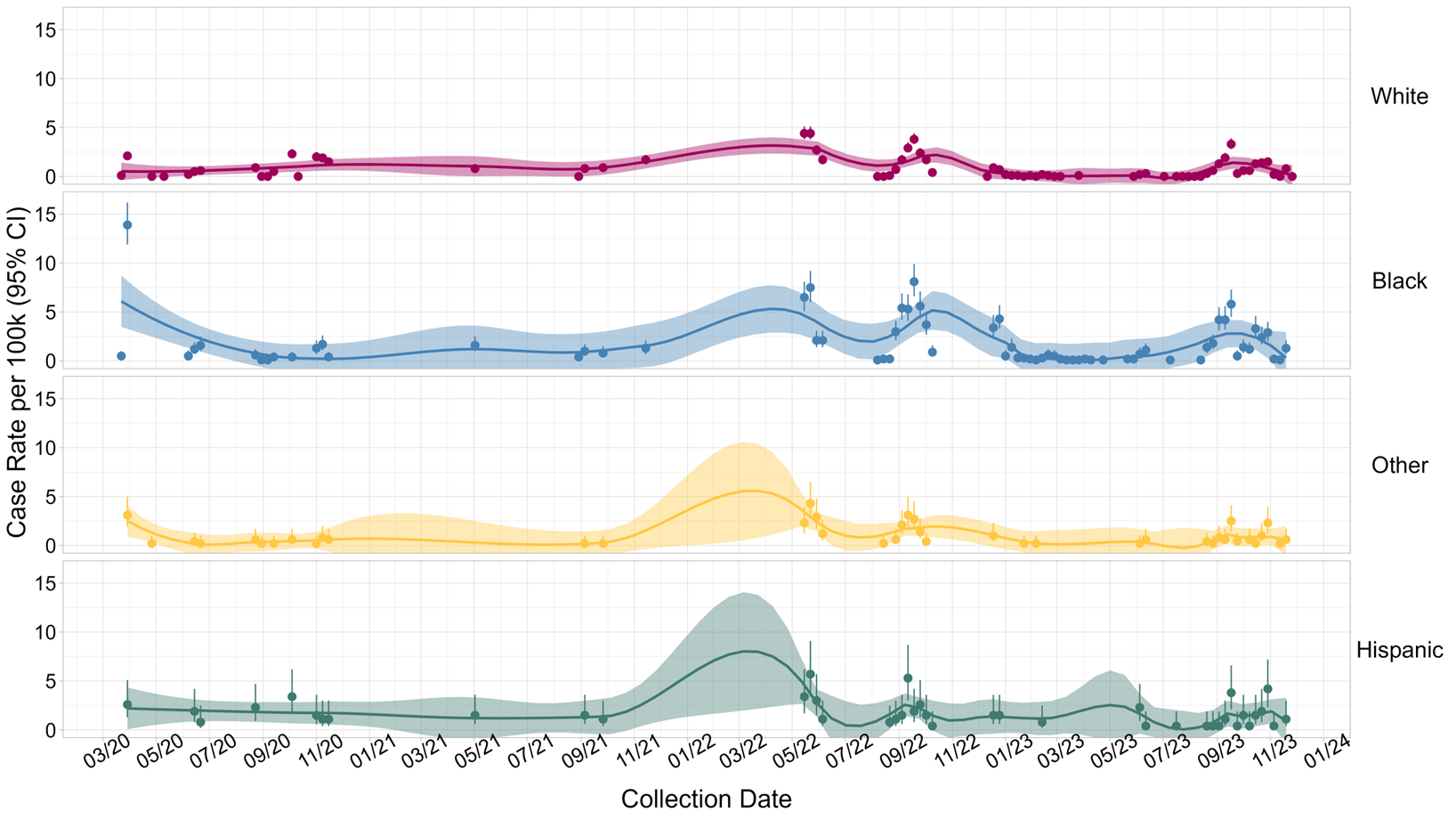


**Figure S5. Weekly COVID-19 Incidence per 100,000 by Race and Ethnicity.** Race/ethnicity-specific weekly COVID-19 incidence per 100,000 population. Race and Hispanic ethnicity are not mutually exclusive. Loess smoothing applied (span=0.25).

**Figure S6:**


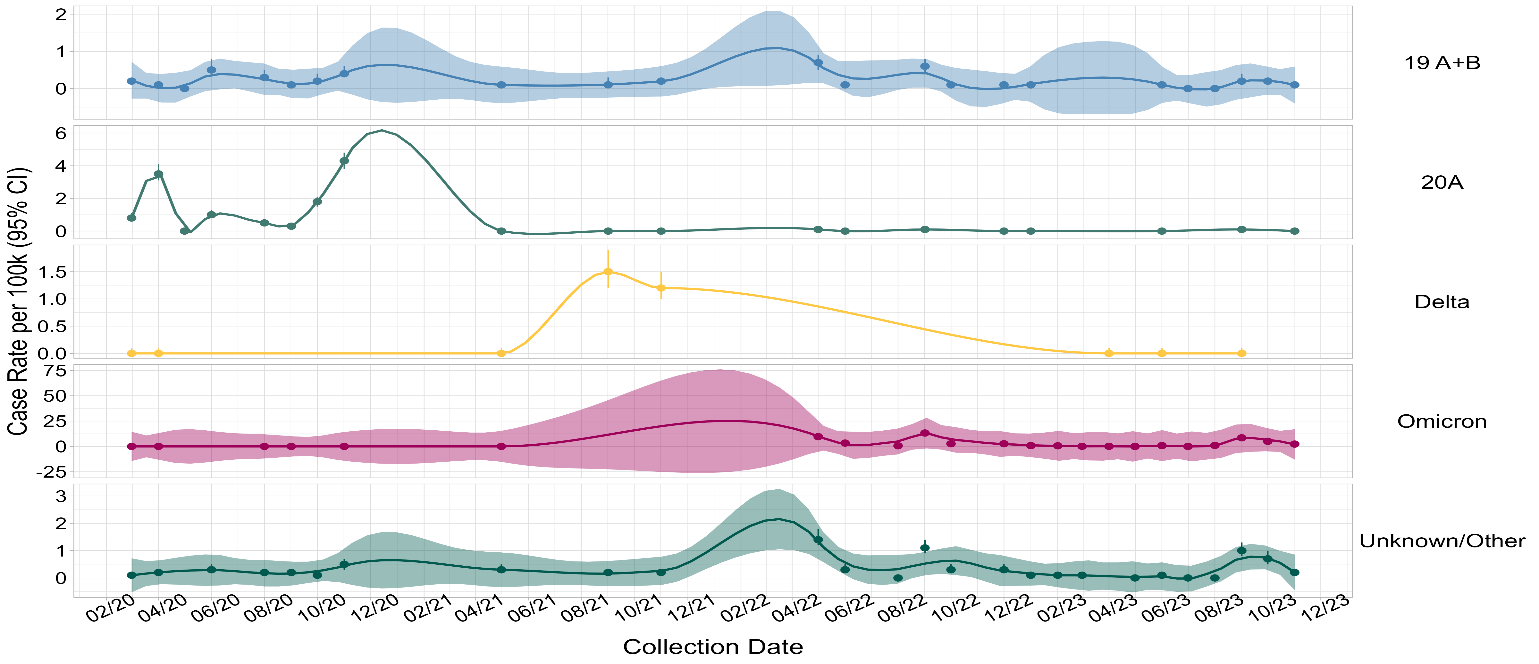


**Figure S6. Variant-Specific Monthly Incidence with Varying Y-Axis.** Varying y-axis dot plot showing monthly incidence by SARS-CoV-2 variant. Loess smoothing applied (span=0.25). Notes: Variant-Specific monthly COVID-19 cases per 100K population with loess smoothing’ with varying y-axes. COVID-19 cases n=4,583; denominator N=5,728,013. Population denominator from American Community Survey 2015-2019; ZCTA-specific totals of 295 Michigan ZIP codes. 95% Confidence interval (CI).

**Figure S7:**


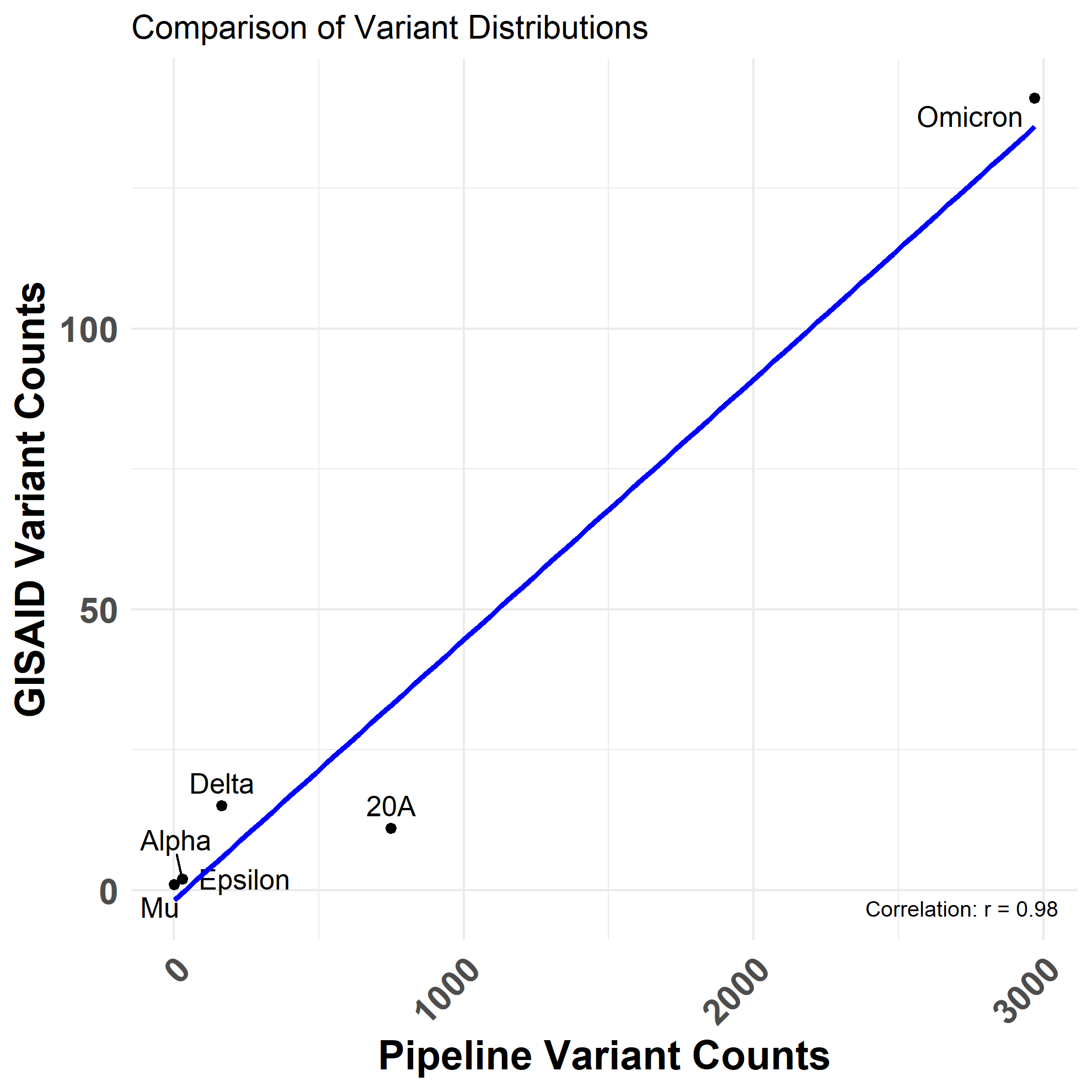


**Figure S7:** **Correlation between GSAID of MI and Genomic Surveillance in Southeast MI.** Correlation Between SARS-CoV-2 Variants Detected by GISAID and Pipeline Samples. The graph shows the correlation between the variants identified by GISAID, based on the number of sequenced cases, and 4583 samples collected from Henry Ford Hospital, r=0.98.

**Figure S8:**
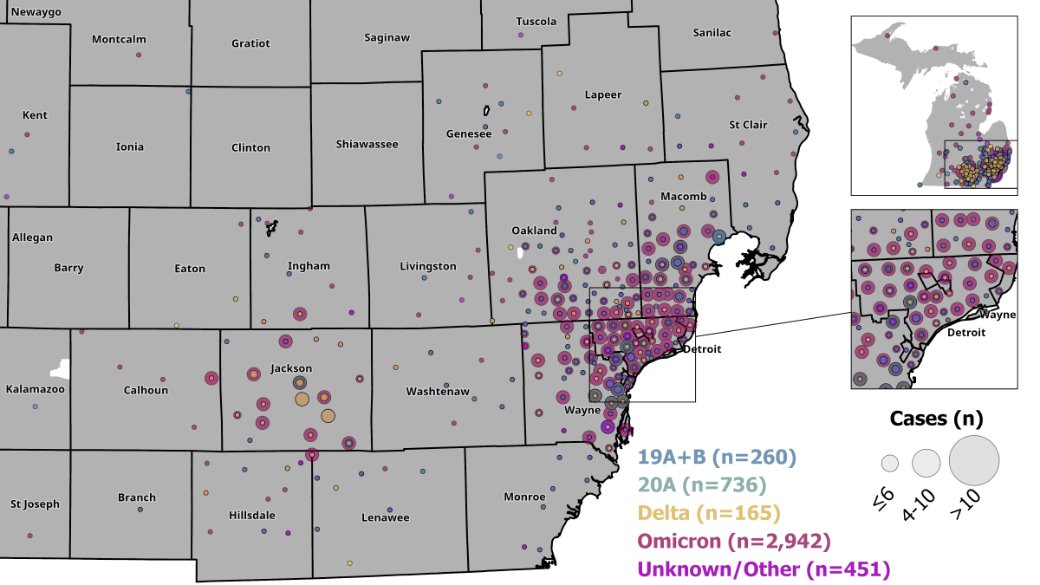


**Figure S8: Variant Distribution Across Southeast MI.** Choropleth map displaying the geographic variant distribution of the total number of COVID-19 cases sequenced in the SIPPHIRE study (n=4,583) by residential ZIP code in Michigan.

**Figure S9:**


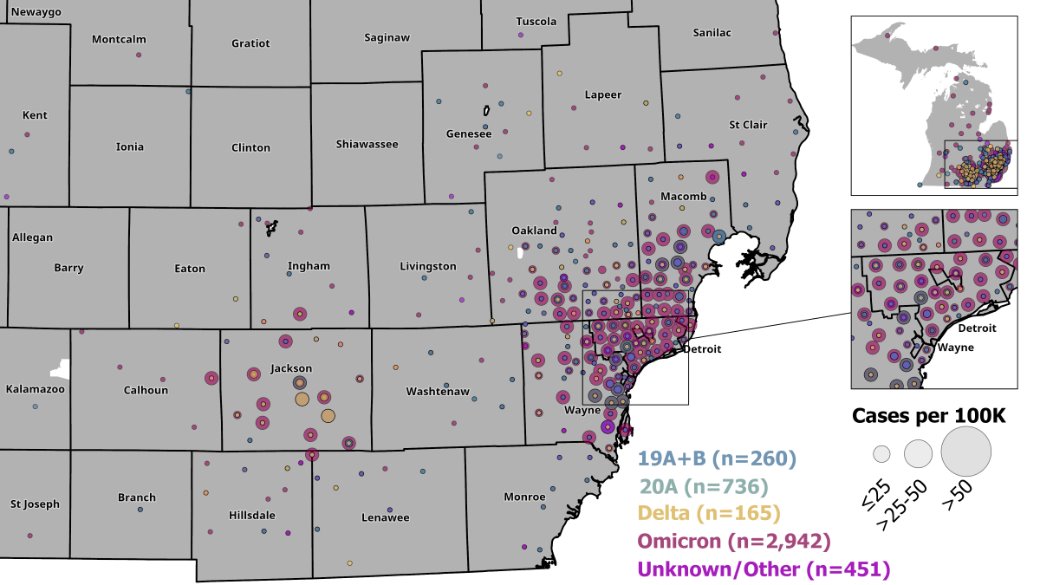


**Figure S9: Variant Prevalence Across Southeast MI.** Choropleth map showing variant prevalence of COVID-19 cases (n=4,583) by residential ZIP code.

**Figure S10:**


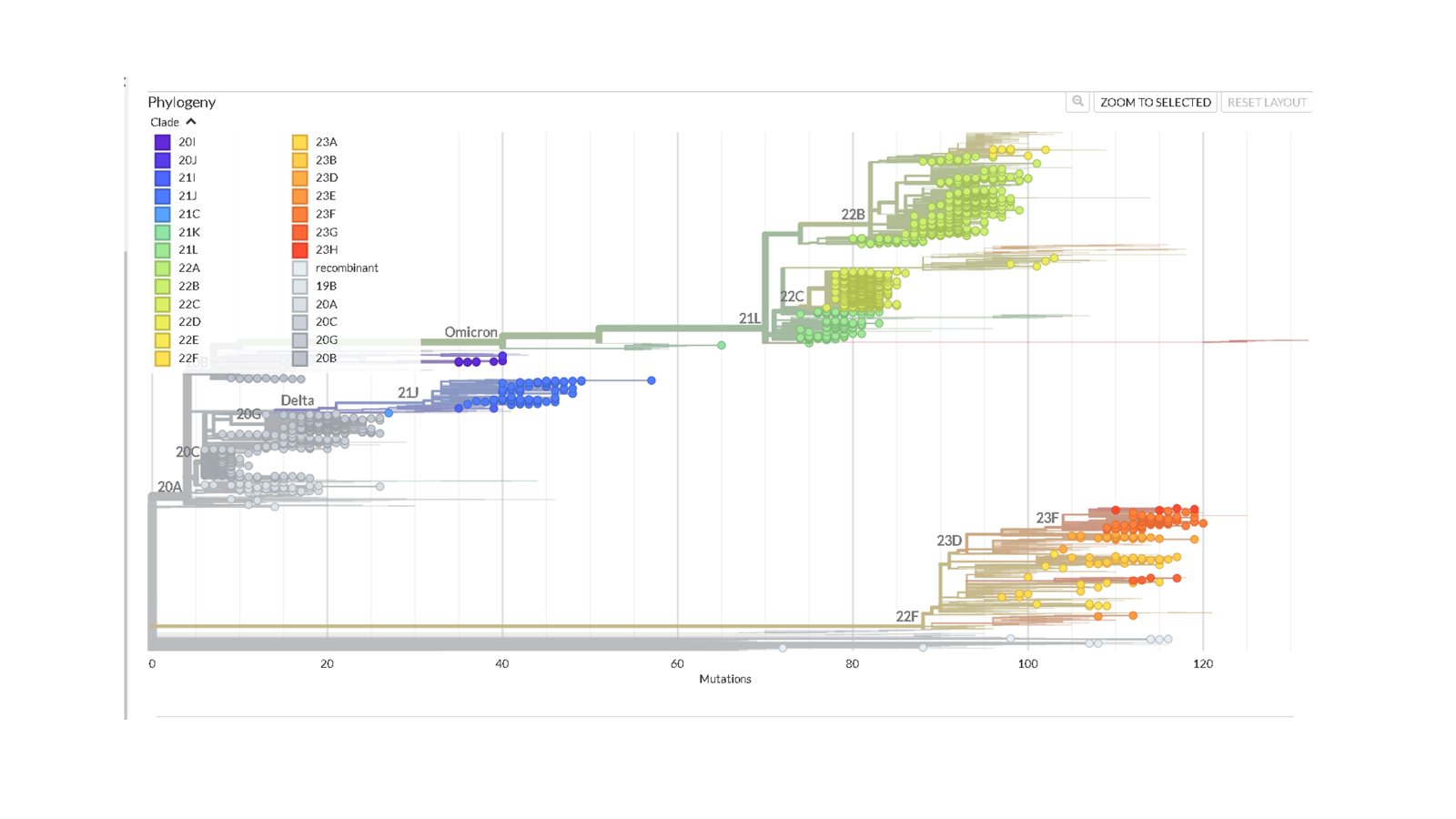
**Figure S10: Phylogenetic Tree for HFH Samples.** Between January 2020 and December 2021, SARS-CoV-2 samples collected from HFH included 79 Delta variants, followed by 10 Alpha and 10 Omicron variants. Approximately 50% of the samples (349 in total) belonged to clade 20C. The other major clades identified were 20G and 21J, comprising 158 and 77 samples, respectively.

**Figure S11:**


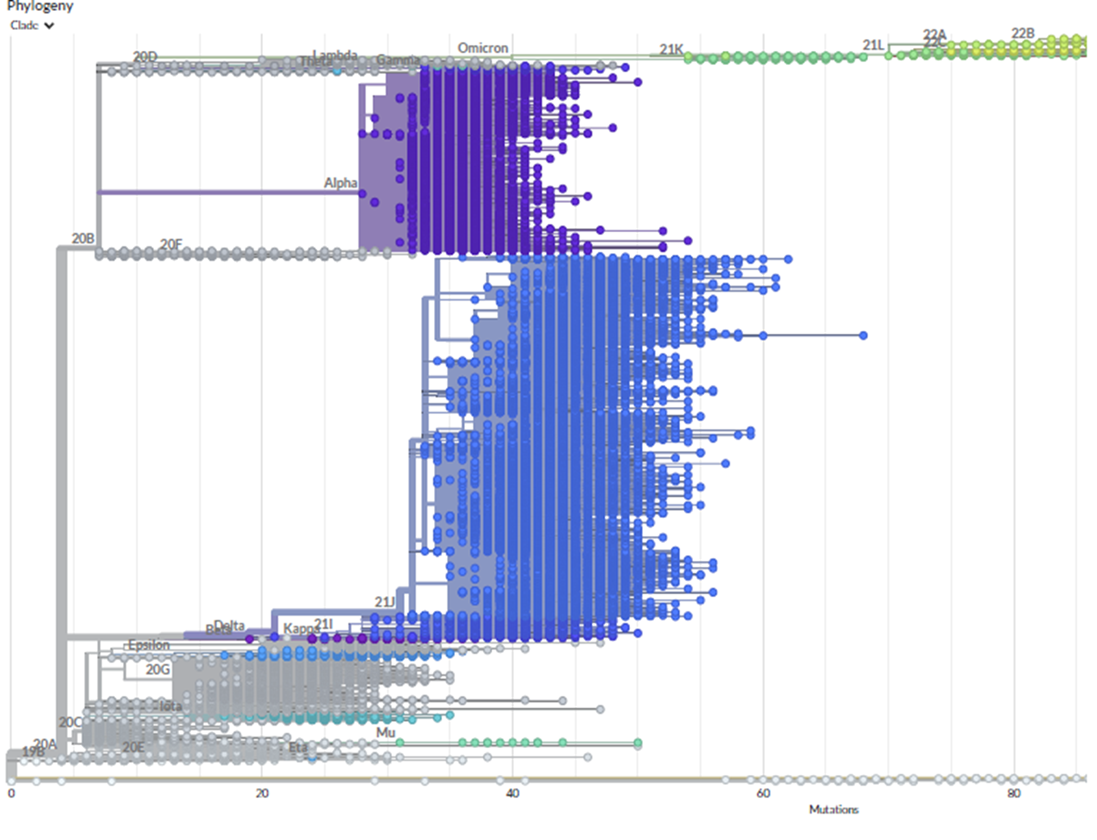


**Figure S11: Phylogenetic Tree for GSAID Samples from MI.** Between January 2020 and December 2021, SARS-CoV-2 samples collected from Michigan included 22,522 Delta variants, 10,704 Alpha variants, 461 Iota variants, and 413 Epsilon variants. The most prevalent clade was 21J, represented by 21,301 samples. Other major clades included 20I (10,704 samples), 20C (2,686 samples), and 20G (2,538 samples). Please refer to figure S11 for variant color key.

**Figure S12:**


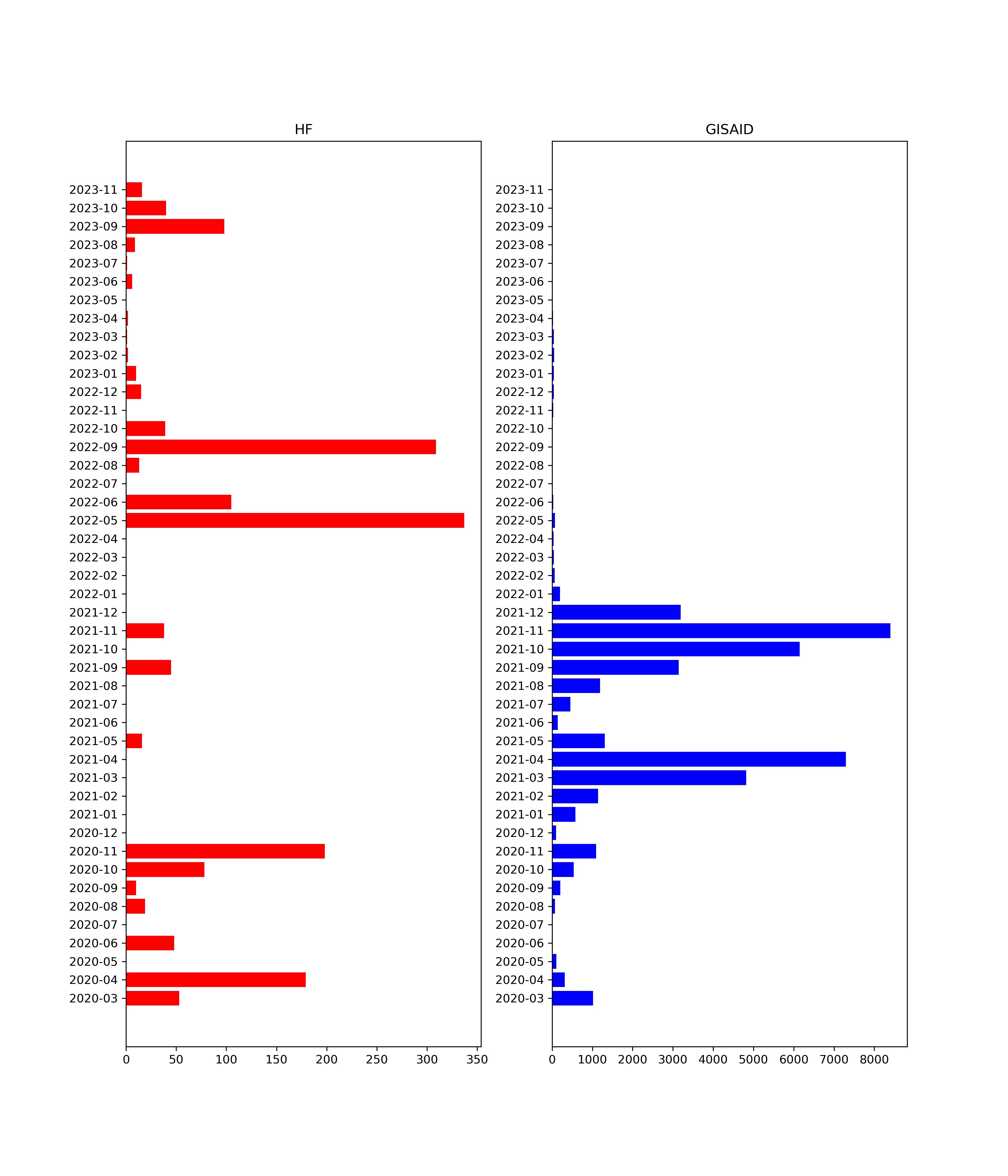


**Figure S12: Comparison of sample distribution between HFH and GISAID.** SARS-CoV-2 genomes submitted from Michigan between January 2020 and December 2021 were retrieved from GISAID for comparison with clinical samples collected from HFH during the same period. Only high-quality genomes, as determined by Nextclade analysis, were included. The monthly distribution of samples differed significantly between HFH and GISAID datasets (p < 0.01).

**Figure S13:**


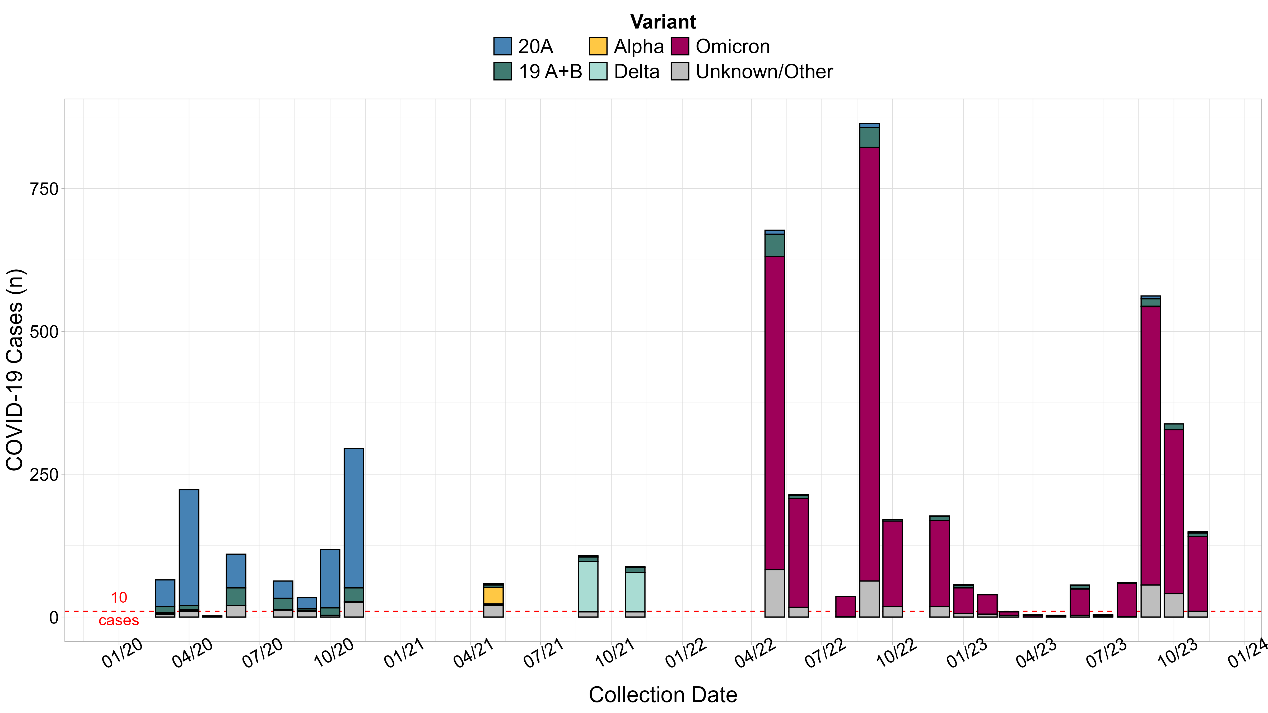


**Figure S13. Monthly COVID-19 Cases by Variant.** Stacked bar chart showing monthly COVID-19 cases (n=4,583), stratified by SARS-CoV-2 variant, among analytic sample (n=4,583 COVID-19 cases).

**Figure S14**:

|  | **Year** | **Month** | **Sample count** |
| --- | --- | --- | --- |
|  |  |  |  |
|  |  |  |  |
| **1** | 2020 | Mar | 66 |
| **2** | 2020 | Apr | 225 |
| **3** | 2020 | May | 2 |
| **4** | 2020 | Jun | 112 |
| **5** | 2020 | Aug | 64 |
| **6** | 2020 | Sep | 35 |
| **7** | 2020 | Oct | 118 |
| **8** | 2020 | Nov | 301 |
| **9** | 2021 | May | 58 |
| **10** | 2021 | Sep | 109 |
| **11** | 2021 | Nov | 90 |
| **12** | 2021 | Dec | 1 |
| **13** | 2022 | May | 686 |
| **14** | 2022 | Jun | 218 |
| **15** | 2022 | Aug | 36 |
| **16** | 2022 | Sep | 876 |
| **17** | 2022 | Oct | 174 |
| **18** | 2022 | Dec | 178 |
| **19** | 2023 | Jan | 58 |
| **20** | 2023 | Feb | 39 |
| **21** | 2023 | Mar | 9 |
| **22** | 2023 | Apr | 4 |
| **23** | 2023 | May | 3 |
| **24** | 2023 | Jun | 57 |
| **25** | 2023 | Jul | 4 |
| **26** | 2023 | Aug | 60 |
| **27** | 2023 | Sep | 564 |
| **28** | 2023 | Oct | 341 |
| **29** | 2023 | Nov | 149 |

**Figure S14. HFH Sample Counts per Month/Year.** Table showing monthly COVID-19 sample counts by month and year among analytic sample (n=4,58).
